# The evolution of human and ape hand proportions

**DOI:** 10.1038/ncomms8717

**Published:** 2015-07-14

**Authors:** Sergio Almécija, Jeroen B. Smaers, William L. Jungers

**Affiliations:** 1 Center for the Advanced Study of Human Paleobiology, Department of Anthropology, The George Washington University, Washington, DC 20052, USA; 2 Department of Anatomical Sciences, Stony Brook University, Stony Brook, New York 11794, USA; 3 Institut Català de Paleontologia Miquel Crusafont (ICP), Universitat Autònoma de Barcelona, Edifici Z (ICTA-ICP), campus de la UAB, c/ de les Columnes, s/n., 08193 Cerdanyola del Vallès (Barcelona), Spain; 4 Department of Anthropology, Stony Brook University, Stony Brook, New York 11794, USA

## Abstract

Human hands are distinguished from apes by possessing longer thumbs relative to fingers. However, this simple ape-human dichotomy fails to provide an adequate framework for testing competing hypotheses of human evolution and for reconstructing the morphology of the last common ancestor (LCA) of humans and chimpanzees. We inspect human and ape hand-length proportions using phylogenetically informed morphometric analyses and test alternative models of evolution along the anthropoid tree of life, including fossils like the plesiomorphic ape *Proconsul heseloni* and the hominins *Ardipithecus ramidus* and *Australopithecus sediba*. Our results reveal high levels of hand disparity among modern hominoids, which are explained by different evolutionary processes: autapomorphic evolution in hylobatids (extreme digital and thumb elongation), convergent adaptation between chimpanzees and orangutans (digital elongation) and comparatively little change in gorillas and hominins. The human (and australopith) high thumb-to-digits ratio required little change since the LCA, and was acquired convergently with other highly dexterous anthropoids.

## Abstract (web summary)

The human hand can be distinguished from that of apes by its long thumb relative to fingers. Here the authors show that hand proportions vary greatly among ape species and that the human hand evolved from an ancestor that was more similar to humans than to chimpanzees.

## Graphical abstract

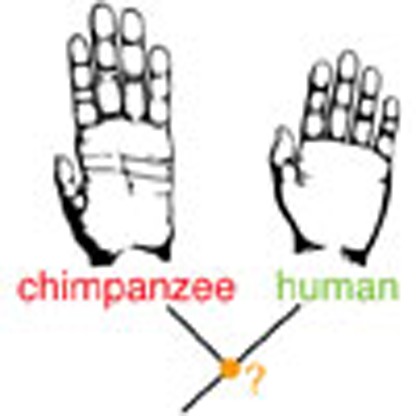

The hand is one of the most distinctive traits of humankind and one of our main sources of interaction with the environment^1^. The human hand can be distinguished from that of apes by its long thumb relative to fingers^1^,^2^,^3^,^4^ (Fig. 1a), which has been related functionally to different selective regimes—manipulation vs locomotion—acting on human and ape hands^1^,^5^. During the first half of the twentieth century, theories on human evolution were dominated by the view that humans split very early from the common stock of apes, and largely preserved generalized (plesiomorphic) hand proportions similar to other anthropoid primates^6^,^7^,^8^. To the contrary, extant apes were seen as extremely specialized animals adapted for below-branch suspension^6^,^7^. However, since the molecular revolution in the 1980–1990s (which provided unequivocal evidence for humans and chimpanzees being sister taxa)^9^ a prevalent and influential evolutionary paradigm—said to be based on parsimony—has assumed that the last common ancestor (LCA) of chimpanzees and humans was similar to a modern chimpanzee (for example, ref. 10). This shift resurrected the ‘troglodytian' stage in human evolution, which assumes that a chimp-like knuckle-walking ancestor preceded human bipedalism (for example, ref. 11). Most subsequent hypotheses dealing with human hand evolution have been framed assuming a ‘long-handed/short-thumbed' chimp-like hand as the starting points of the LCA and basal hominins, with strong selective pressures acting to reverse these proportions in the context of stone tool-making and/or as a by-product of drastic changes in foot morphology in the human career (for example, ref. 12). However, the current fossil evidence of early hominins^2^,^5^,^13^,^14^ and fossil apes^15^,^16^,^17^,^18^ challenges this paradigm. Collectively these fossils suggest instead that hand proportions approaching the modern human condition could in fact be largely plesiomorphic^2^,^4^,^13^, as was previously suggested before the advent of molecular phylogenetics. If that were the case, this would have profound implications relevant to the locomotor adaptations of the chimpanzee-human LCA, as well as the relationship between human hand structure and the origins of systematized stone tool culture.

To address this complex discussion, and to provide a deeper understanding on the evolution of the human and ape hand, in this study we perform a stepwise series of detailed morphometric and evolutionary analyses on the hand-length proportions of modern apes and humans, as compared with a large sample of extant anthropoid primates and key fossils preserving sufficiently complete associated hands. This fossil sample is constituted by the early hominins *Ardipithecus ramidus* (4.4 Myr ago)^2^ and *Australopithecus sediba* (∼2 Myr ago)^14^, as well as the primitive African ape *Proconsul heseloni* (∼18 Myr ago)^15^ and the European fossil great ape *Hispanopithecus laietanus* (9.6 Myr ago)^17^. First, we inspect thumb length relative to the lateral digits (as revealed by ray four; that is, intrinsic hand proportions) to show that humans are distinctive from apes for this important functional measure, but not from some other anthropoids. Second, we analyse individual hand elements as proportions adjusted via overall body size (that is, extrinsic hand proportions) to test whether modern apes—and more especially African great apes—represent a homogeneous group from which humans depart. Here we further show that modern hominoids constitute a highly heterogeneous group with differences that cannot be explained by phylogenetic proximity or size-related effects. Third, we enlist phylogenetically informed comparative methods to map how the evolution of hand-length proportions has played out along the individual lineages of our comparative sample. These methods employ statistical models that establish principles of how continuous trait change is likely to have unfolded over time, and we explore those principles to infer how the variation observed in comparative trait measurements is likely to have changed along the individual branches of a (independently derived molecular-based) phylogeny. Importantly from a statistical viewpoint, these methods allow the comparative data (including the fossils) to be analysed within an alternative-hypothesis-testing framework that assesses the statistical fit of alternative evolutionary scenarios. In our case, we determine how hand-length proportions changed over time and quantify the relative likelihood support of alternative evolutionary hypothesis, thus providing a novel and rigorous analysis of human and ape hand evolution.

Our results reveal that the different hand morphologies exhibited by modern hominoids reflect different evolutionary processes: hylobatids display an autapomorphic hand due to extreme digital and thumb elongation; chimpanzees and orangutans exhibit convergent adaptation related to digital elongation (to a lesser degree than hylobatids); whereas the gorilla and hominin lineages experienced little change by comparison (that is, their overall hand proportions are largely plesiomorphic within catarrhines). These results support the view that the long thumb relative to fingers characterizing the human (and australopith) hand required little change since the chimpanzee-human LCA, and was acquired in convergence with other highly dexterous anthropoids such as capuchins and gelada baboons.

## Results

### Intrinsic hand proportions

**Figure 1:**
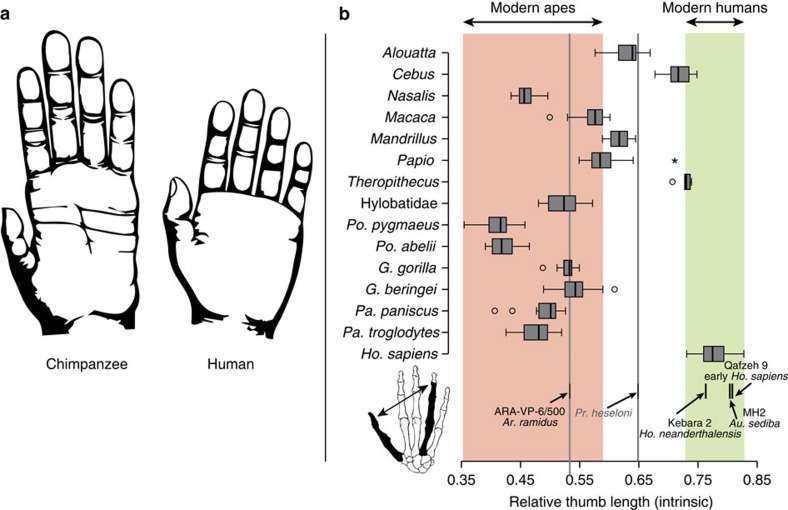
Intrinsic hand proportions of humans and other anthropoid primates. (**a**) Drawings of a chimpanzee and human hands are shown to similar scale. (**b**) Relative length of the thumb=pollical/fourth ray lengths (minus distal fourth phalanx; see inset). Box represents the interquartile range, centerline is the median, whiskers represent non-outlier range and dots are outliers. The ranges of humans and modern apes are highlighted (green and red-shaded areas, respectively). Samples for each boxplot are *Homo sapiens* (*n*=40), *Pan troglodytes* (*n*=34), *Pan paniscus* (*n*=12), *Gorilla beringei* (*n*=21), *Gorilla gorilla* (*n*=13), *Pongo abelii* (*n*=8), *Pongo pygmaeus* (*n*=19), Hylobatidae (*n*=14), *Theropithecus* (*n*=5), *Papio* (*n*=50), *Mandrillus* (*n*=3), *Macaca* (*n*=18), *Nasalis* (*n*=14), *Cebus* (*n*=11) and *Alouatta* (*n*=8). The values for *Pr. heseloni* and *Ar. ramidus* are projected onto the remaining taxa to facilitate visual comparisons.

Hand proportions of humans are usually compared with those of apes using the thumb-to-digit ratio (or IHPs), which is a good functional measure of thumb opposability and therefore a proxy for manual dexterity (for example, refs 1, 14, 19). Accordingly, we queried our anthropoid sample (see details of our sample in Supplementary Table 1) to see whether our IHP measure (as revealed by the thumb-to-fourth ray ratio; Fig. 1b) was consistent with previous observations that humans can easily be distinguished from modern apes by a long thumb relative to the other digits^4^,^5^,^14^. The modern human IHP range is well above that of modern apes (that is, no overlap; analysis of variance (ANOVA) with Bonferroni *post hoc* comparisons, *P*<0.001; see Supplementary Table 2 for details on the taxa-specific comparisons), which can be linked directly to the human capability (unique among modern hominoids^20^) to perform an efficient ‘pad-to-pad precision grasping' (that is, broad contact of the distal pads of the thumb and index finger, Supplementary Note 1)^1^,^4^,^5^,^13^. In contrast, chimpanzees and especially orangutans are found to have significantly shorter thumbs than gorillas and hylobatids (ANOVA with Bonferroni *post hoc* comparisons, *P*<0.001). Fossil hominins fall within the modern human range, but *Ar. ramidus* exhibits a shorter thumb (within the gorilla-hylobatid range), implying limits to its precision grasping capabilities. Most non-hominoid anthropoids, including the fossil ape *Pr. heseloni*, exhibit IHP ranges in-between modern apes and humans. Both *Cebus* and *Theropithecus* overlap in this index with humans, supporting the relationship between this ratio and enhanced manipulative skills (see Supplementary Note 1).

### Extrinsic hand proportions

**Figure 2:**
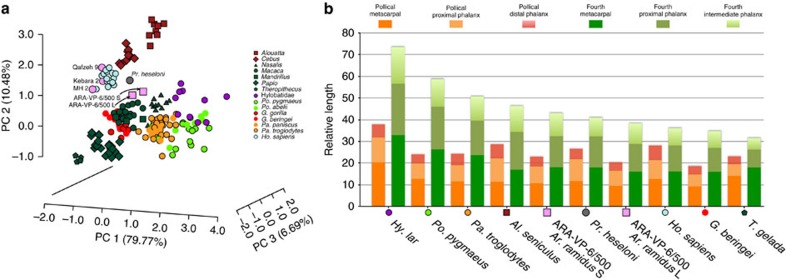
Extrinsic hand proportions of humans and other anthropoid primates. (**a**) Principal components analysis of the body mass-adjusted hand lengths. (**b**) Summary of the contribution of each hand element in selected anthropoids. Species are arranged by maximum length of ray IV (notice that the thumb does not follow the same trend). ARA-VP-6/500 L refers to an iteration of *Ar. ramidus* with an estimated body mass of 50.8 kg, whereas ARA-VP-6/500 S uses a smaller estimate of 35.7 kg.

Despite the aforementioned functional connections, IHPs provide limited information regarding what distinguishes humans from apes: is it a longer thumb, shorter digits or a combination of both? More specifically, which elements contribute most to the overall ray length? To clarify this and inspect how each of the individual elements of the thumb and ray IV contribute to IHPs (Fig. 1b), we standardized each length relative to overall body size (approximated by the cube root of its body mass, BM), creating relative length shape ratios of external hand proportions (EHPs; Supplementary Fig. 1). Major trends of EHP variation between the individuals in our anthropoid sample are summarized and inspected by means of principal components analysis of extant and fossil individuals (Supplementary Table 3), revealing high EHP heterogeneity in extant hominoids (and in non-hominoid anthropoids; Fig. 2a, Supplementary Fig. 1). In other words, there is a clear EHP structure that allows the characterization of the hominoid taxa. Statistical differences in EHP between each great ape genus, hylobatids and humans were established (*P*<0.001) by means of multivariate analysis of variance (MANOVA with Bonferroni-corrected *post hoc* pairwise comparisons; see Supplementary Table 4). Differences among extant great ape genera are more apparent when the eigenanalysis is carried out exclusively on great ape individuals (Supplementary Fig. 3), even revealing significant differences between species of gorillas (*P*=0.014) and chimpanzees (*P*=0.047). EHPs of selected species are depicted to help understand extreme morphologies along the major axes of variation in shape space (Fig. 2b). A complex pattern is revealed: hylobatids, orangutans and chimpanzees (in this order) exhibit longer digits than humans, but gorillas do not. Thumb length follows a rather different trend: hylobatids have both the longest digits and the longest thumbs, whereas *Theropithecus* displays the shortest digits but not the shortest thumbs (rather, eastern gorillas do). For *Ar. ramidus* we inspect two different relative shape possibilities based on substantially different but plausible BM estimations: 50.8 kg (as a quadruped) and 35.7 kg (as a biped). Fossil hominins display a modern human pattern, but *Ar. ramidus* shows only slightly longer or shorter (BM-depending) digits than *Pr. heseloni* (that is, it is intermediate between humans and chimpanzees), but in both cases it exhibits shorter thumbs (specifically shorter pollical phalanges; Supplementary Table 3) than this fossil ape and other hominins, and occupies a different region of EHP shape space (Fig. 2 and Supplementary Fig. 2). The observed differences in EHP between hominoid taxa cannot be merely attributed to size-dependent effects (that is, allometry; Supplementary Fig. 4, Supplementary Table 5).

### The evolution of human and ape hand proportions

**Figure 3:**
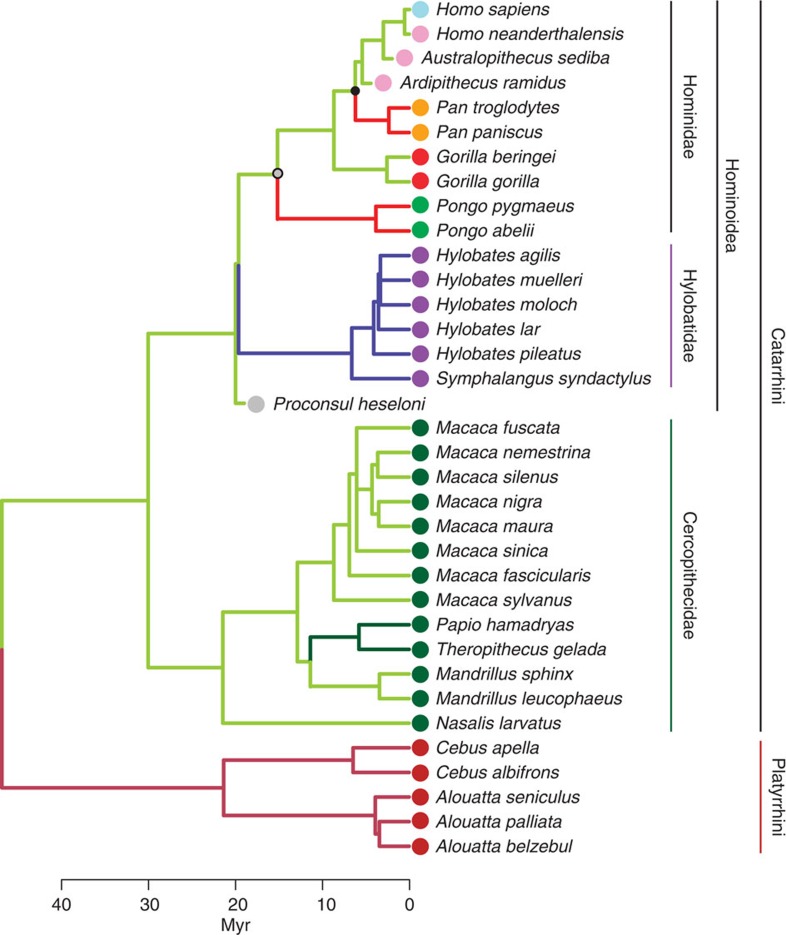
Time-calibrated phylogenetic tree showing the estimated adaptive regimes in our anthropoid sample. Adaptive optima are based on the two major axes of extrinsic hand proportions (EHP) variation between extant and fossil species (accounting for 94.5% of the variation). Branches are colour-coded according to different adaptive regimes (revealing that *Pan* and *Pongo* -red edges- are convergent). Clades are colour-coded (circles) as follows: brown, platyrrhines; dark green, cercopithecids; purple, hylobatids; light green, orangutans; red, gorillas; orange, chimpanzees; pink, fossil hominins; light blue, modern humans. The nodes corresponding to the last common ancestor (LCA) of great apes-humans and chimpanzees-humans are highlighted.

**Figure 4:**
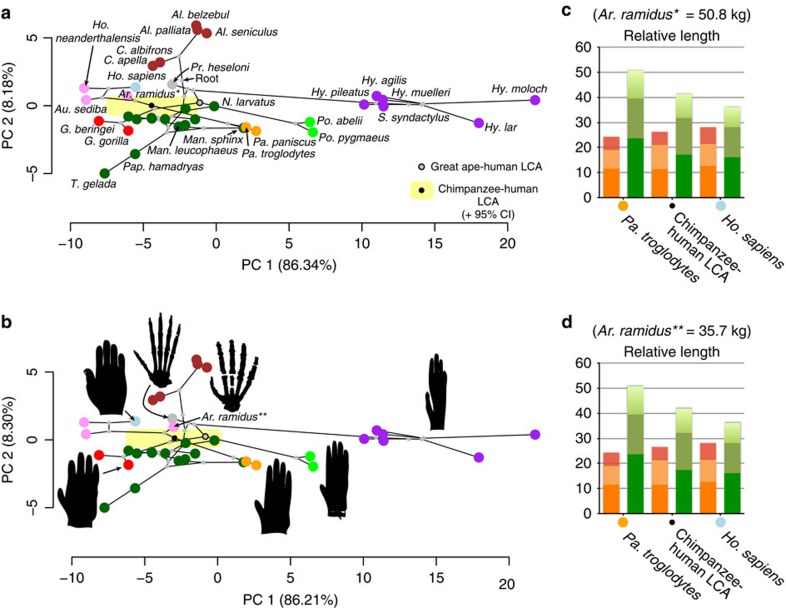
The evolutionary history of human and ape hand proportions. Phylomorphospace projection of the phylogeny presented in Fig. 3 onto the two first principal components (PCs) of extrinsic hand proportions (EHP) in extant and fossil species. Taxa are colour-coded as in the phylogenetic tree; internal nodes (that is, ancestral-states reconstructed using maximum likelihood) are also indicated, highlighting the positions in shape space of the great ape-human and chimpanzee-human LCAs (plus 95% confidence intervals for the latter estimate). (**a**) EHP of *Ardipithecus ramidus* estimated using 50.8 kg. Owing to space constrictions, macaque species are not labelled. (**b**) Iteration using 35.7 kg for *Ar. ramidus*. Outlines (scaled to similar length) of extant and fossil apes and *Ar. ramidus* are plotted in this phylomorphospace to help visualizing major shape changes occurred during ape and human hand evolution. Panels (**c**) and (**d**) depict the EHP of chimpanzees and humans vis-à-vis their reconstructed last common ancestor (LCA) assuming, respectively, 50.8 kg and 35.7 kg for *Ar. ramidus*.

Previous observations on modern ape thoraces and limbs suggest that living apes show similar but not identical adaptations to accommodate similar functional demands related to specialized climbing and suspension (especially *Pan* and *Pongo*), reinforcing the role of parallelism in ape evolution^3^,^21^,^22^, a phenomenon explained by common evolutionary developmental pathways in closely related taxa^23^. To test this homoplastic hypothesis for similarities in hand-length proportions between suspensory taxa, we enlist the ‘surface' method^24^, which allows inferring the history of adaptive diversification in hominoids (and other anthropoids) using a phylogeny (Fig. 3) and phenotypic data, in this case the two major axes of EHP variation among extant and fossil species (accounting for 94.5% of variance; see Fig. 4 and Supplementary Table 7). This method models adaptive evolutionary scenarios by fitting a multi-regime Ornstein Uhlenbeck (OU) stabilizing selection model^25^ to the tip data. This procedure allows taxonomic units to undergo shifts towards different phenotypes (‘adaptive peaks') and can be used to identify cases where multiple lineages have discovered the same selective regimes (that is, convergence). Regimes are here understood as comprising a group of taxonomic units that are inferred to have similar phenotypes. Adaptive peaks can be understood as the optimal phenotypic values that characterize the different regimes. The advantage of the surface method is that it locates regime shifts without a prior identification of regimes. The method hereby fits a series of stabilizing selection models and uses a data-driven stepwise algorithm to locate phenotypic shifts on the tree. Thus, this method allows to ‘naively' detect instances of phenotypic convergence in human and ape hand proportions. Starting with an OU model in which all species are attracted to a single adaptive peak in morphospace, ‘surface' uses a stepwise model selection procedure based on the finite-samples Akaike information criterion (AICc)^26^,^27^ to fit increasingly complex multi-regime models. At each step, a new regime shift is added to the branch of the phylogeny that most improves model fit across all the variables inspected, and shifts are added until no further improvement is achieved. To verify true convergence, this method then evaluates whether the AICc score is further improved by allowing different species to shift towards shared adaptive regimes rather than requiring each one to occupy its own peak. For the EHPs, ‘surface' detects five adaptive optima (see edge colours in phylogenetic tree in Fig. 3) corresponding to (1) *Cebus* and *Alouatta*; (2) *Papio* and *Theropithecus*; (3) *Macaca, Mandrillus, Nasalis, Gorilla* and hominins; (4) hylobatids; and (5) *Pan* and *Pongo*. In other words, in terms of human and great ape evolution ‘surface' identifies convergent evolution between the EHPs of *Pan* and *Pongo*, whereas *Gorilla* and hominins share a more plesiomorphic condition for catarrhines. To verify this result, we compare the statistical fit of this evolutionary scenario with that of five other evolutionary hypotheses based on the respective relative AICc weights (Supplementary Fig. 5; Supplementary Table 8). The alternative models include Brownian motion evolution, a single-regime OU model, a multi-regime OU model differentiating the different clades, and most importantly an alternative version of the five-regime OU model detected by ‘surface' in which the condition shared by *Pan* and *Pongo* is hypothesized to represent the plesiomorphic state for great apes (OU5 ‘alt' in Supplementary Fig. 5). Our results support the ‘surface' output as the best fit model using either a large or a small body size estimate for *Ar. ramidus* (ΔAIC_c_=0.00, AIC_c_ weight=1.00), and even when excluding *Ar. ramidus* and *Pr. heseloni* from the analysis (ΔAIC_c_=0.00, AIC_c_ weight=0.77). To test the sensitivity of our results to a possible sampling bias due to the higher number of hominoid species in comparison with monkey clades in our sample, we repeat the analysis once more after excluding the most speciose and morphologically derived group of hominoids (the hylobatid species), together with the fossil closest to the hominoid LCA in our sample (that is, *Pr. heseloni*). Again, ‘surface' identifies a best fit model in which *Pan* and *Pongo* are convergent, with the difference that the slightly reduced digits of gorillas and hominins are now interpreted as being convergent with baboons, while the remaining monkey taxa share a common, more plesiomorphic, regime (Supplementary Fig. 6). This evolutionary scenario also has the best support (ΔAIC_c_=0.00, AIC_c_ weight=0.92) when compared with Brownian motion, and four other alternative evolutionary scenarios (Supplementary Table 8). Importantly in terms of human and ape evolution, irrespective of the difference in results between the full vs reduced hominoid sample, the similarities between the EHP of hominins and gorillas are reconstructed as representing the plesiomorphic condition for the African ape and human clade (Fig. 3), while *Pan* would be more derived (and convergent with *Pongo*).

Furthermore, to visually track major evolutionary changes driving differences between apes and humans, we summarize the evolutionary history of hominoid hand length diversification (as compared with platyrrhine and cercopithecid monkey out-groups) by means of a phylomorphospace approach^28^. These are the steps that we followed: First, we reconstructed hypothetical ancestral morphologies (that is, internal nodes in Fig. 3) using a maximum likelihood approach and plotted them on the shape space defined by the two major EHP axes of variation among extant and fossil species (Fig. 4). Second, we mapped our time-calibrated phylogenetic tree (Fig. 3) onto this shape space by connecting the ancestral sate reconstructions and the terminal taxa. The lengths and orientations of the branches of this phylomorphospace allows one to intuitively visualize the magnitude and directionality of inferred shape changes along each branch of the tree. Owing to the possible impact of *Ar. ramidus* in the reconstruction of the chimpanzee-human LCA (based on its proximity in time), we present this analysis with both large and small body size estimates (Fig. 4a,b respectively), as well as by excluding *Ar. ramidus* and *Pr. heseloni* (Supplementary Fig. 7). In all cases, major evolutionary changes along PC1 (∼86% of variance; see Supplementary Table 7) relate to digital (primarily metacarpal and proximal phalanx) lengthening/shortening (positive and negative values, respectively), whereas PC2 (∼8% of variance) relates to thumb proximal phalanx (positive values) and digital metacarpal (negative values) lengthening, and thereby serves to separate our platyrrhine and catarrhine taxa (especially baboons). Although the position of *Ar. ramidus* in shape space differs depending on estimated BM, the overall evolutionary pattern remains constant: from moderate digital length, digital lengthening has been achieved to different degrees and independently in chimpanzees, orangutans and hylobatids (in this increasing order; with *Pan* and *Pongo* sharing the same adaptive optimum, see Fig. 3). In contrast, hominins and gorillas (especially eastern gorillas) have slightly reduced their digital lengths (although both would still represent the same evolutionary regime, see Fig. 3). In terms of thumb evolution, only a modest reduction in extant great apes and slight elongation in later hominins appears to have occurred. It is worth noticing that, irrespective of which *Ar. ramidus* BM estimate is used, *Pan* falls clearly outside of the 95% confident interval for the estimated chimpanzee-human LCA, whereas *Ar. ramidus* is very close to it (Fig. 4), as previously suggested^2^,^29^. This supports the idea that chimpanzees exhibit derived hands, in this case convergent with *Pongo* (Fig. 3).

This previous phylogenetic patterning observed in our EHP morphospace (that is, homoplasy along PC1, and more clade-specific groups along PC2; see Fig. 4) was tested with Blomberg's *K* statistic^30^. Our results indicate that for PC2 variance is concentrated among clades (*K*>1; 1,000 permutations, *P*=0.001): *Alouatta* (long thumb proximal phalanx and short digital metacarpal) and baboons (reverse condition of howler monkeys) are situated at opposite extremes, and other cercopithecids and hominoids exhibit intermediate values. For PC1, however, the variance is concentrated within clades (*K*<1; 1,000 permutations, *P*=0.001), indicating that the observed variance in finger length (that is, PC1) is larger than expected based on the structure of the tree. This supports the idea of adaptive evolution (that is, shape change associated with change in function)^31^ in hominoid finger length uncorrelated with phylogeny^30^. In other words, finger lengthening has been achieved homoplastically in different ape lineages (probably in relation to increased suspensory behaviours), as also revealed by our multi-regime OU modelling (Fig. 3, Supplementary Figs 5 and 6) and phylomorphospace approach (Fig. 4).

**Figure 5:**
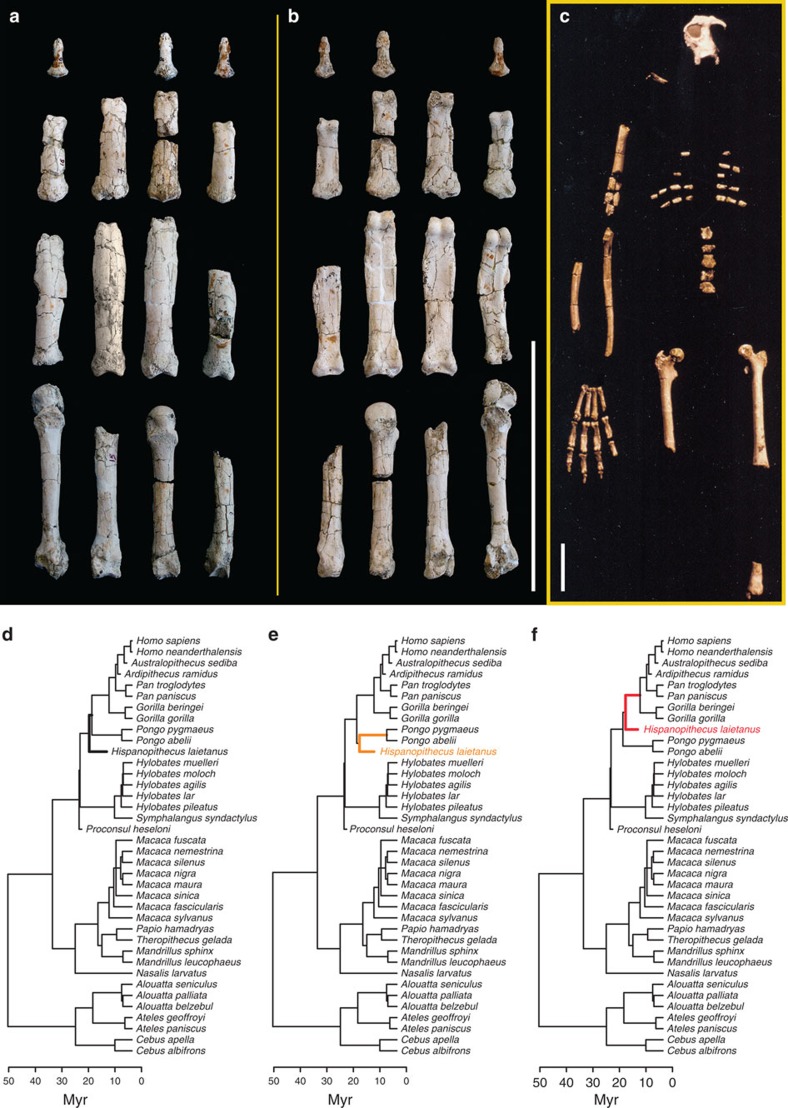
The hand of the late Miocene ape *Hispanopithecus laietanus*. Its reconstructed hand is displayed in dorsal (**a**) and palmar (**b**) views, and together with its associated skeleton (**c**). This species represents the earliest specialized adaptations for below-branch suspension in the fossil ape record^33^, although its hand combining short metacarpals and long phalanges, dorsally oriented hamato-metacarpal and metacarpo-phalangeal joints, presents no modern analogues^17^. The phylogenetic position of *Hispanopithecus* is still highly debated: stem great ape (**d**), stem pongine (**e**) or stem hominine (**e**)? Scale bars represent 10 cm. Reconstruction of the IPS 18800 (*Hispanopithecus*) skeleton in panel (**c**) reproduced with the permission of Salvador Moyà-Solà and Meike Köhler.

**Figure 6:**
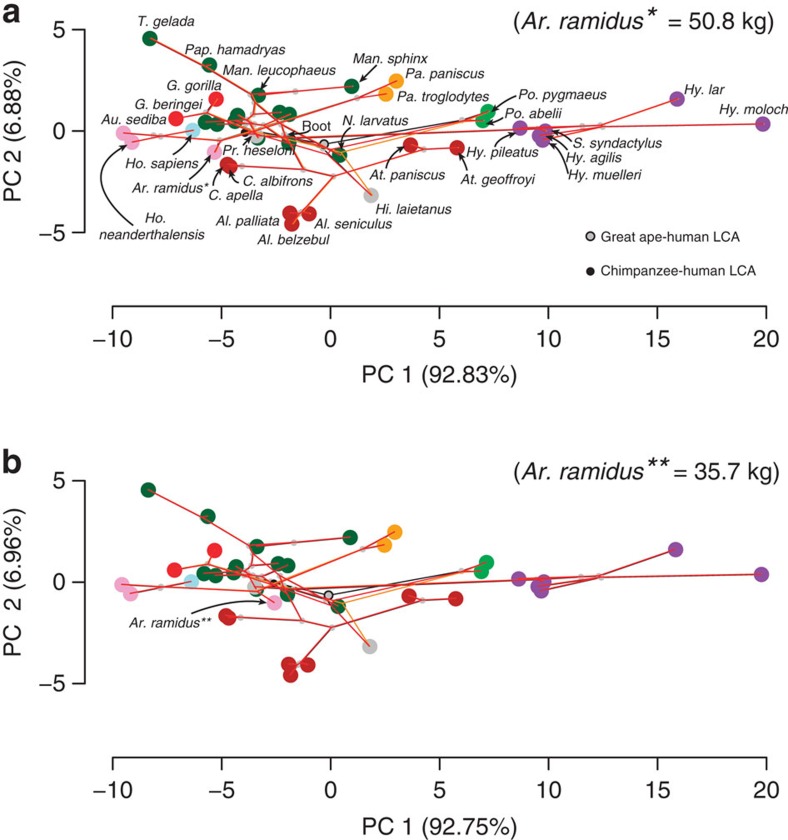
Reconstructed evolutionary histories of human and ape digital extrinsic proportions. The phylomorphospace approach was limited to the three long bones of ray IV to include the fossil ape *Hispanopithecus laietanus* and *Ateles* species. The same analysis was iterated with the large (**a**) and small (**b**) body mass estimates of *Ardipithecus ramidus* (finding no differences in the overall evolutionary pattern). Internal nodes (that is, ancestral-state reconstructions) and branch lengths are indicated for three different phylogenetic hypotheses: *Hi. laietanus* as a stem great ape (black), a stem pongine (orange) and stem African ape (red). Species names are indicated in (**a**) with the exception of macaques.

To inspect how the addition of more taxa with long fingers affects our evolutionary reconstructions of digital length, we revisit the phylomorphospace after excluding the thumb elements. Specifically, we incorporate the fossil ape *Hispanopithecus laietanus*^17^ (which does not preserve thumb elements; Fig. 5a–c) and the suspensory platyrrhine *Ateles* (which exhibits only a vestigial thumb^32^)*. Hi. laietanus* represents the earliest evidence of specialized adaptations for below-branch suspension in the fossil ape record^17^,^33^. However, its phylogenetic position is not resolved, being alternatively considered as a stem great ape, a stem pongine or even a stem hominine (Fig. 5d–f). In the fourth ray morphospace (Fig. 6), PC1 (∼92% of variance; Supplementary Table 7) is mainly related positively to metacarpal and proximal phalanx lengths, whereas PC2 (∼6% of variance) is positively related to metacarpal length and negatively to proximal phalanx length. When ancestral state reconstructions and phylogenetic mapping are inspected in this phylomorphospace, the overall evolutionary pattern reflecting homoplasy in modern (and fossil) ape digital elongation is also evident, irrespective of the BM estimate of *Ar. ramidus* and the phylogenetic position of *Hi. laietanus* (Fig. 6). Specifically, these results also indicate independent digital elongation (to different degrees) in hylobatids, orangutans, chimpanzees, spider monkeys and *Hi. laietanus*. Although chimpanzees and *Hi. laietanus* exhibit a similar relative digital length (Supplementary Fig. 4b), it has been achieved by different means. In contrast to chimpanzees and baboons that display long metacarpals relative to proximal phalanges (as revealed by PC1 in Fig. 6), *Hi. laietanus* approaches a condition similar to that of howler monkeys by exhibiting long phalanges relative to short metacarpals (as revealed by PC2 in Fig. 6). Overall, these results match the previously recognized mosaic nature of the *Hi. laietanus* hand morphology^17^, which suggests that its suspensory-related adaptations evolved independently from that of other apes. More broadly, even though the living hominoid lineages represent the few remnants of a much more prolific group during the Miocene^22^, the evidence presented above indicate that hominoids constitute a highly diversified group in terms of hand proportions (as identified in Fig. 2, Supplementary Fig. 1, and Figs 4 and 6).

Finally, we reconstruct the evolution of IHPs (see Fig. 1) of humans and modern apes as having evolved in opposite directions from moderate IHP similar to those exhibited by *Pr. heseloni* (Supplementary Fig. 8). On the basis of the previous results on EHP evolution (Fig. 4), this implies that the relatively long thumb of humans and short thumb of modern apes would have been driven primarily by digital elongation/shortening rather than by drastic changes in thumb length. The comparison of eight multi-regime OU models (Supplementary Table 8) identifies a best fit model (ΔAIC_c_=0.00, AIC_c_ weight=1.00) based on four different optima in which *Cebus* and *Theropithecus* are convergent with *Australopithecus*/*Homo* for a relatively long (that is, easily opposable) thumb; *Pan* is convergent with *Pongo* and *Nasalis* for very short thumbs; and hylobatids, gorillas and *Ar. ramidus* share the putative plesiomorphic, ‘moderate' condition for crown apes (Supplementary Fig. 9).

## Discussion

Collectively, our results support several evolutionary scenarios with profound and far-reaching implications regarding ape and human origins (see Supplementary Note 2 for an extended background in this matter): (1) extant apes are heterogeneous in terms of hand-length proportions (as inspected by means of their EHP; Fig. 2, Supplementary Figs S1–S3), a finding contrary to a *Pan-*like ancestor ‘based on parsimony'. In other words, our results falsify the view that extant apes, and particularly African apes, constitute a homogeneous group with subtle deviations from a similar allometric pattern (for example, ref. 34; see also our Supplementary Fig. 4). This previous idea, together with the phylogenetic proximity between *Pan* and *Homo*, has been commonly used as support for the hypothesis that hominins evolved from a *Pan*-like ancestor (for example, ref. 10). Our results, and the palaeontological evidence indicating mosaic-manner evolution of the hominoid skeleton^16^,^17^,^33^, should caution us against relying on evolutionary scenarios that assume that extant apes are good ‘overall' ancestral models^22^. (2) Low levels of inter-limb integration in hominoids relative to other anthropoids (that is, higher postcranial heterogeneity) have been used to claim that during hominoid evolution natural selection operated for functional dissociation between homologous pairs of limbs, allowing for evolutionary ‘experimentation'^35^. For hand length proportions, our results indicate that *Pan* and *Pongo* are convergent (Fig. 3, Supplementary Fig. 9), whereas hylobatids evolved long digits in parallel to them, but to a larger extent (PC1 in Figs 4 and 6), thus representing extreme outliers (related to their small size and specialized ricochetal brachiation). Thus, in terms of evolution of digital elongation, we hypothesize that in some ape lineages natural selection acted on (co)variation in inter-limb lengths and hand proportions in the context of specialized adaptation for below-branch suspension. This scenario matches previous evidence suggesting the extant ape lineages survived the late Miocene ape extinction event because they specialized, and were able to share habitats with the radiating and soon to be dominant cercopithecids^23^,^36^. (3) Similarities in hand proportions between humans and gorillas and our ancestral African ape reconstruction (Figs 2, 3, 4) indicate that the possession of very long digits was not a requisite for the advent of knuckle walking. (4) These similarities also indicate that specialized tree climbing was not precluded in australopiths based on hand length. (5) Humans have only slightly modified finger and thumb lengths since their LCA with *Pan* (Fig. 4, Supplementary Fig. 8), probably in relation to refined manipulation, as suggested by the convergent similarities with *Cebus* and *Theropithecus* (Fig. 1, Supplementary Figs 8 and 9). This probably occurred with the advent of habitual bipedalism in hominins, and almost certainly preceded regular stone culture^4^,^5^,^13^.

Our results provide a detailed picture on the evolution of the hand that is drawn from a multiple-regime model-fitting approach that infers the evolutionary scenario that indicates the optimal statistical fit for the observed differences in hand proportions between apes and humans, in terms of both the total amount and direction of shape changes. These results are also most consistent with previous observations on pervasive homoplasy and complex evolution of the modern ape postcranium^3^,^21^,^35^, as well as with the available evidence from fossil apes and early hominins^1^,^2^,^22^,^29^,^37^.

## Methods

### Intrinsic hand proportions

The IHPs were computed as the ratio between the long bones of the thumb (metacarpal, proximal and distal phalanges) and the long bones of the fourth ray but excluding the distal phalanx, which is not well represented in the fossil record (that is, metacarpal, proximal and intermediate phalanges). A total of 270 modern anthropoids, including humans, all the species of great apes, hylobatids, as well as cercopithecid and platyrrhine monkeys (Supplementary Table 1) were compared with available fossils (Fig. 1), and differences between extant taxa were tested via ANOVA (with Bonferroni *posthoc* comparisons; Supplementary Table 2). As the emphasis of this work is on the evolution of the human hand, comparisons were made to our closest living relatives (that is, the great apes) at the species level. Hylobatids were pooled at the family level and extant non-hominoid anthropoids at the genus level. Some of the monkey groups are represented by small samples (for example, *Theropithecus, Mandrillus*) due to the difficulty of finding associated distal phalanges (pollical in this case) in the museum collections (most of them were apparently lost during the skinning and preparation process). However, we still included these taxa because they provide relevant phylogenetic background to understanding the evolution of hand proportions in apes and humans.

The fossil sample included the associated hands of *Ar. ramidus* (ARA-VP-6/500) and *Au. sediba* (MH2), whose measurements were taken from published sources^2^,^14^; the hands of *Homo neanderthalensis* (Kebara 2) and fossil *Homo sapiens* (Qafzeh 9), which were measured from the originals, and the fossil ape *Proconsul heseloni*, measurements of which were also taken from the originals (KNM-KPS 1, KNM-KPS 3 and KNM-RU 2036). For *Ar. ramidus*, pollical proximal phalanx length in ARA-VP-6/500 was estimated in 25.7 mm from the pollical proximal phalanx/fourth metacarpal proportion in the ARA-VP-7/2 individual, and the fourth metacarpal length in ARA-VP-6/500, as in Lovejoy *et al.*, 2009 (ref. 2). For *Pr. heseloni*, the estimated length of the KNM-RU 2036 pollical metacarpal was extracted from the literature^15^,^38^. IHP in *Pr. heseloni* was computed from the mean proportions obtained after standardizing each manual element by the BM in the three specimens (see next section).

### Shape analyses of extrinsic hand proportions

EHPs were computed for an extant sample of 187 anthropoid primates (Supplementary Table 1) and the fossils described above by standardizing the length (in mm) of each of the six manual elements (inspected in the IHP) by cube root of the BM (kg) associated with each individual. As tissue density is very similar in all terrestrial organisms (and closely approaches unity), mass can be taken as roughly equivalent to volume, and the cube root of BM (‘the nominal length of measure') is therefore proportional to linear ‘size'^39^,^40^,^41^.

Major trends in EHP variation between the individuals of our sample were examined by means of a principal components analysis carried out on the covariance matrix (Fig. 2; Supplementary Figs 2 and 3; Supplementary Table 3). Differences between groups were tested via MANOVA (and Bonferroni *post hoc* comparisons; Supplementary Table 4) of the first three PCs. EHPs were further examined for the fourth ray only (if thumb bones are missing) to include the late Miocene ape *Hispanopithecus laietanus* (IPS 18800; Figs 5 and 6), for which manual lengths were taken from the original fossil^17^. As this latter analysis was restricted to the fourth ray, we also included species of *Ateles,* which shows a ‘rudimentary' thumb^32^.

### Allometric regressions

We relied on ratios to assess intrinsic and extrinsic hand proportions in our sample, and thus quantify the actual shape of each individual as a scale-free proportion. We favour ratios here over residuals because residuals derived from allometric regressions are not a property inherent to the individuals, but rather are sample-dependent^42^. However, to test whether differences between the hand length proportions in our ape sample could be attributed to size-related shape changes (that is, allometry), we constructed separate bivaritate plots for the natural log-transformed lengths of the thumb and fourth ray relative BM (Supplementary Fig. 5). Least square regressions were fitted to these data independently for the extant hominid genera and hylobatids, and grade shifts were inspected through Bonferroni *post hoc* comparisons between estimated marginal means (Supplementary Table 5) after checking for homogeneity of slopes via analysis of covariance (ANCOVA).

### Body mass estimation

Known BMs (kg) were taken from museum records for the extant samples whenever available. Individuals with recorded BM were used to derive genus-specific regressions of BM on femoral head diameter (FHD; in mm). These equations were then used to estimate the BM of additional individuals of unknown BM from their FHD (for example, the *Pan-*specific regression was used to estimate the BM of *Pan* specimens only). Generic regressions are provided in Supplementary Table 6.

We also derived our own BM estimates for fossils. For example, we used a regression of BM on FHD of sex-specific means of a diverse group of ‘small humans' (Supplementary Table 6) to estimate the BM of *Au. sediba*; this yielded a value of 32.5 kg, close to the previous (slightly higher) estimate based on the calcaneus^43^, but slightly lower than a previous estimate based on FHD^44^. The case of *Ar. ramidus* is more complex: first, a published FHD is not available for this species, although estimated FHD can be bracketed from acetabular diameter^45^ as approximately 32–37 mm; second, since *Ar. ramidus* is described as a facultative biped still practicing above-branch pronograde quadrupedalism^2^,^37^, the most appropriate reference sample (bipeds versus quadrupeds) for estimates of its BM is open to question (see also Sarmiento and Meldrum for a different interpretation)^46^. Accordingly, we estimated the BM in ARA-VP-6/500 twice using alternative regressions based on chimpanzees (the hominoid quadrupedal reference sample) and the aforementioned ‘small humans' (the bipedal training sample), which yielded values of 50.8 and 35.7 kg, respectively. For *Hispanopithecus laietanus* (IPS 18800) BM estimates using a *Pongo* or a *Pan* regression generate very similar results (36.9 and 37.6 kg, respectively); therefore, an average of these two values was used, which is comparable to previous estimates^33^. For other hominin fossils, a BM estimate based on FHD was available in the literature for Qafzeh 9 (ref. 47), and another prediction based on bi-iliac breadth was used for Kebara 2 (ref. 48). For the *Proconsul heseloni* individuals, BM estimates using different methods and regressions from various preserved anatomical regions were also available^49^.

### Phylogenetic trees

The time-scaled phylogeny used in this work is based on a consensus chronometric tree of extant anthropoid taxa downloaded from 10kTrees Website (ver. 3; http://10ktrees.fas.harvard.edu/), which provides phylogenies sampled from a Bayesian phylogenetic analysis of eleven mitochondrial and six autosomal genes available in GenBank, and adding branch lengths dated with fossils^50^. With the exception of Neanderthals (for which molecular data is available), other fossil species were introduced *post hoc*. For these fossil species, as a criterion of standardization, ghost lineages of one million years were added to the published age of the fossil. *Au. sediba* and *Ar. ramidus* were introduced into the hominin lineage as it is most commonly accepted^29^,^51^, although controversy exists for *Ar. ramidus*^52^,^53^,^54^. *Pr. heseloni* is most universally interpreted as a stem hominoid^15^,^16^,^55^,^56^, although others consider it as a stem catarrhine^52^. There is not general consensus for placement of the late Miocene ape from Spain *Hi. laietanus*. Its phylogenetic position is debated between stem great ape^57^, stem pongine^33^ or stem hominine^55^. Therefore, we created three different trees including this taxon and reiterated the analyses (Figs 5 and 6).

### Multi-regime OU modelling

Based on its mathematical tractability, the most frequently used statistical model of evolution is Brownian motion, which assumes that traits change at each unit of time with a mean change of zero and unknown and constant variance^58^,^59^,^60^. Within Brownian motion, the evolution of a continuous trait ‘X' along a branch over time increment ‘*t*' is quantified as d*X*(*t*)=*σ*d*B*(*t*), where ‘*σ*' constitutes the magnitude of undirected, stochastic evolution (‘*σ*2' is generally presented as the Brownian rate parameter) and ‘d*B*(t)' is Gaussian white noise. Although novel phylogenetic comparative methods continue using Brownian evolution as a baseline model, they incorporate additional parameters to model possible deviations from the pure gradual mode of evolution assumed by Brownian motion. Ornstein–Uhlenbeck (OU) models incorporate stabilizing selection as a constraint and hereby quantify the evolution of a continuous trait ‘*X*' as d*X*(*t*)= *α*[*θ*−*X*(*t*)]d*t*+*σ*d*B*(*t*) where ‘*σ*' captures the stochastic evolution of Brownian motion, ‘*α*' determines the rate of adaptive evolution towards an optimum trait value ‘*θ*' (see ref. 25). This standard OU model has been modified into multiple-optima OU models allowing optima to vary across the phylogeny^61^. In these implementations the parameters are defined *a priori*, which allows testing the relative likelihood of alternative parameterizations (whereby each parameterization characterizes a different evolutionary scenario that explains the evolution of a trait). Importantly, this approach allows fitting multivariate data, circumventing issues that stem from iteratively fitting univariate data. These model-fitting approaches are available in the *R* package ‘ouch' (ref. 62) and are particularly powerful in testing the relative likelihood of alternative evolutionary scenarios explaining multivariate data. Although this OU model fitting approach comprises a powerful way of comparing the likelihood of alternative evolutionary scenarios, it leaves open the possibility that the ‘best-fit' evolutionary scenario is not included in the research design. In this context, Ingram & Mahler (ref. 24) expanded the OU model fitting approach by developing a way to estimate the number of shifts and their locations on the phylogeny, rather than *a priori* assuming them. This method (‘surface') was developed specifically to identify instances of convergent evolution and can be used to extract the evolutionary scenario that indicates the best statistical fit (that is, the lowest Akaike information criterion based on the finite samples, AICc)^26^,^27^ between the phylogeny and the observed measurements. We subsequently translated the best fit model from ‘surface' to ‘ouch' to compare it with alternative hypotheses in a fully multivariate framework.

### Phylomorphospace

The phylomorphospace approach allows one to visualize the history of morphological diversification of a clade and infer the magnitude and direction of shape change along any branch of the phylogeny^28^. Thus, we reconstructed the evolutionary history of extrinsic hand proportions in apes and humans (and other anthropoid primates) by projecting our phylogenetic trees (Fig. 3; Fig. 5d,e,f) into our morphospaces (=shape space; Figs 4 and 6), based on eigenanalyses of the covariance matrices of the species means (Supplementary Table 7). This was accomplished by reconstructing the position of the internal nodes (that is, ancestral states) using a maximum likelihood (ML) method for continuous characters^63^,^64^. For an evolutionary model based on normally distributed Brownian motion^58^,^59^,^60^ the ML approach yields identical ancestral state estimates to the squared-change parsimony method accounting for branch length, which minimizes the total amount shape change along all the branches of the tree^65^,^66^. In our case, our results including (Fig. 4) and not including (Supplementary Fig. 7) key fossils, or using different phylogenetic positions of the same fossils (Fig. 6) were essentially unchanged. This suggests that, although fossils are useful to more accurately bound ancestral state reconstructions^67^, in our case the overall evolutionary patterns recovered are robust. These visualizations were computed using the *R* package ‘phytools' (ref. 68). 95% confidence intervals (95% CIs) for the last common ancestor (LCA) of chimpanzees and humans were computed using the ‘fastAnc' function implemented in ‘phytools', and are based on equation [6] of Rohlf (ref. 69) that computes the variance on the ancestral states estimates. Once these variances are known, 95% CIs on the estimates can be computed as the estimates +/−1.96 × the square root of the variances.

### Phylogenetic signal

Phylogenetic signal is generally defined as the degree to which related species resemble each other^60^,^70^. We relied on Blomberg's *K* statistic^30^ to assess the amount of phylogenetic signal relative to the amount expected for a character undergoing Brownian motion. This statistic is based on a comparison of the mean squared error of the tip data (measured from the phylogenetic mean) with the mean squared error of the data calculated using the variance-covariance matrix of the tree. This ratio reflects whether the tree accurately describes the variance-covariance pattern in the data, and is subsequently compared with the expected ratio given the size and the shape of the tree (resulting in the *K* statistic). When *K*<1, close relatives resemble each other less than expected under Brownian motion, thus indicating that variance is concentrated within clades rather than among clades. *K*<1 is suggestive of a mode of evolution that departs from pure Brownian motion. This departure from Brownian motion could be caused, among others, by adaptive evolution uncorrelated with the phylogeny (that is, homoplasy). *K*∼1 indicates that the variance in the tips accurately reflects phylogenetic relatedness (a mode of evolution aligning with Brownian motion). When *K*>1, close relatives resemble each other more than expected under Brownian motion (possibly reflecting stabilizing selection). *K* is also a measure of the partitioning of variance. Thus, (with Brownian motion as reference) whether *K*>1 the variance tends to be between clades, whereas if *K*<1 the variance tends to be within clades (Liam Revell, personal communication). The statistical significance of *K* was evaluated with the permutation test (1,000 iterations) described by Blomberg *et al.* (ref. 30).

## Additional information

**How to cite this article**: Almécija, S. *et al.* The evolution of human and ape hand proportions. *Nat. Commun.* 6:7717 doi: 10.1038/ncomms8717 (2015).

## Supplementary Material

Supplementary Information Supplementary Figures 1-9, Supplementary Tables 1-8, Supplementary Notes 1-2 and Supplementary References
