# Supplementary material for: The evolution of human and ape hand proportions
Source: Nat Commun. 2015 Jul 14;6:7717. doi: 10.1038/ncomms8717 (PMC4510966; doi:10.1038/ncomms8717)
Supplement: Supplementary Information — Supplementary Figures 1-9, Supplementary Tables 1-8, Supplementary Notes 1-2 and Supplementary References [file ncomms8717-s1.pdf]

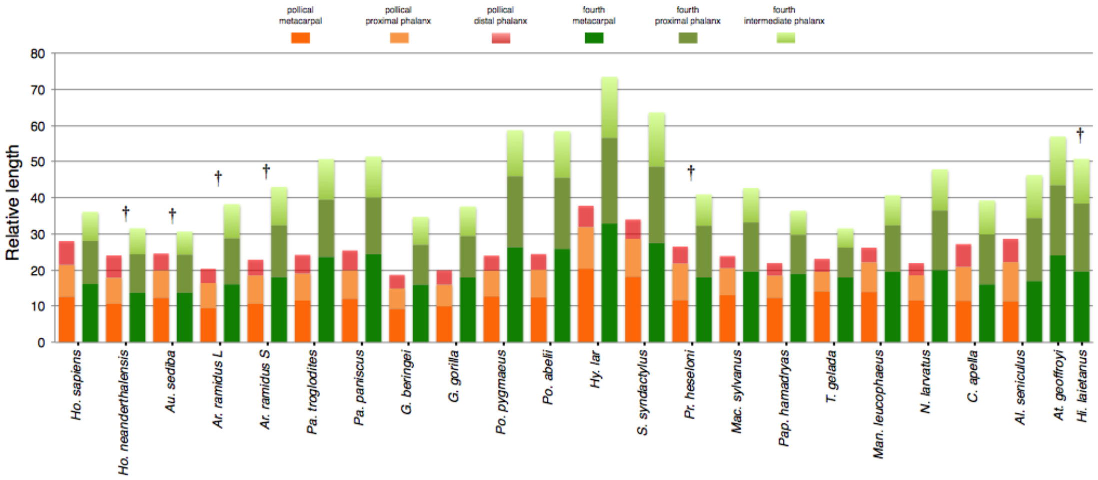

Supplementary Figure 1 | Extrinsic hand proportions of humans, apes and other anthropoid primates. Species means are displayed for all extant hominid species, selected hylobatids, and one species representative of each non-hominoid anthropoid genus. Each element length (in mm) has been adjusted by the known or estimated cube root of body mass (in kg) of the individual. This plot demonstrates the huge disparity in hand proportions among extant hominoids. In comparison to humans, chimpanzees have longer digits and slightly shorter thumbs; contrarily, gorillas exhibit similar digital length but shorter thumbs; orangutans display longer fingers (longer than chimpanzees) and slightly shorter thumbs; finally, hylobatids exhibit much longer digits *and* thumbs. Other relevant observations in non-hominoids: *Theropithecus* approaches the human intrinsic hand proportions (Fig. 1b) by a different mechanism (longer pollical and digital metacarpals but much shorter phalanges). *Nasalis* exhibits chimpanzee-like digital length with a shorter thumb, but African colobines (not included) would exhibit even shorter ones (they display vestigial thumb elements). Fossil species are indicated (†).

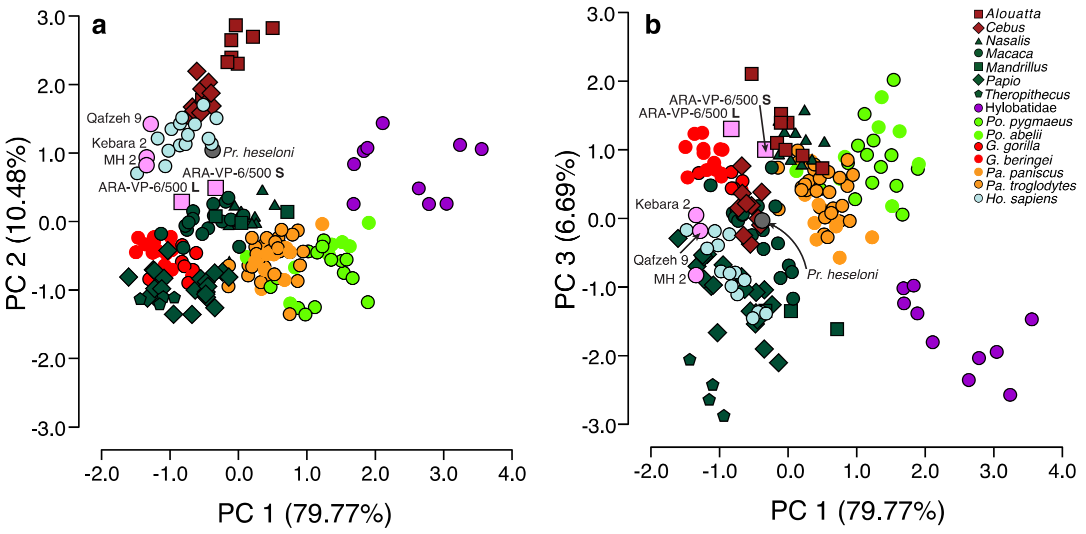

Supplementary Figure 2 | Principal components analysis of extrinsic hand proportions in humans, apes and other anthropoid primates. The results displayed in the three-dimensional plot of Figure 2a are depicted here in two dimensions. **a**, Principal component 1 (PC1) and PC2. **b**, PC1 and PC3. For *Ardipithecus ramidus*, the two body mass-dependent iterations were introduced in the analysis as different operational taxonomic units: ARA-VP-6/500 **L** (50.8 kg) and ARA-VP-6/500 **S** (35.7 kg). PC1 (79.77% of variance; Supplementary Information section 3.3) is related especially to digital length (all elements of ray fourth in this case), and its opposite extremes are represented by the hominins, gorillas and baboons (short digits) on one hand and hylobatids (very long digits) on the other. PC2 (10.48% of variance) is positively related to pollical phalangeal length; hominins (with the exception of both iterations of *Ar. ramidus*), hylobatids, *Proconsul heseloni*, and especially platyrrhines exhibit longer pollical phalnages than extant great apes and especially baboons (exhibiting the shortest thumbs). PC3 (6.69% of variance) is negatively related to pollical metacarpal length; hominins (again with the exception of *Ar. ramidus*), but especially baboons and hylobatids exhibit longer pollical metacarpals than great apes. Each great ape genus, hylobatids and humans exhibit statistical differences in EHP (P<0.001; MANOVA, Supplementary Table 4).

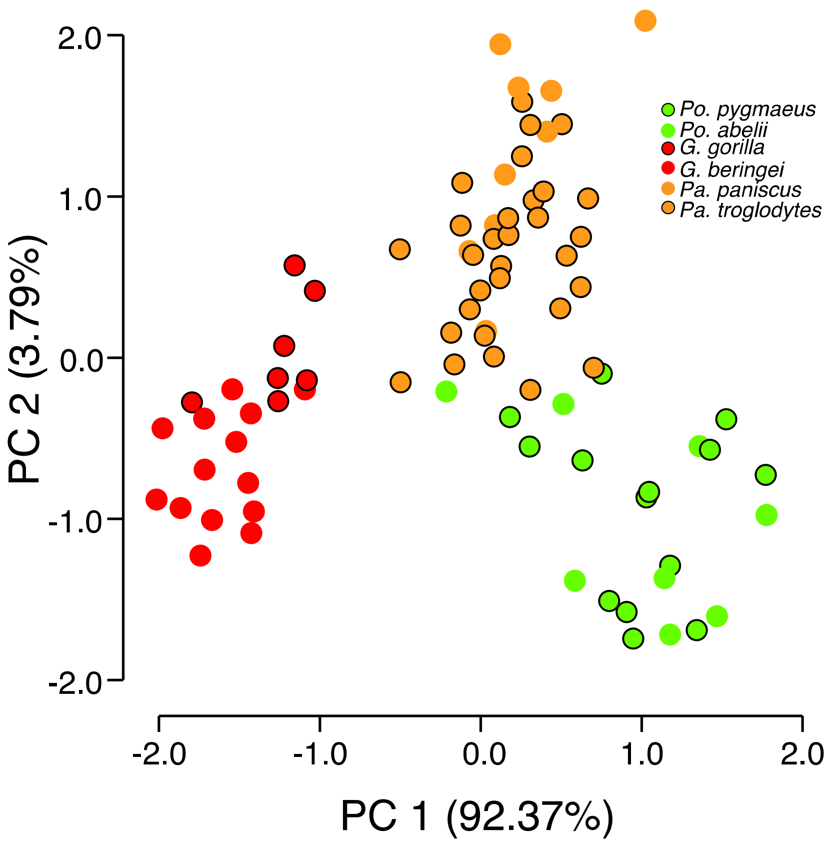

Supplementary Figure 3 | Principal components analysis of extrinsic hand proportions in extant great ape species. The combination of the two first principal components (accounting for >96% of total shape variance; Supplementary Table 3) distinguishes the three extant genera (with just a slight overlap between *Pan* and *Pongo*). PC1 (92.37% of variance) is related to overall length of digit fourth and thumb (excluding the pollical distal phalanx) and completely separates gorillas (short digits and thumb) from chimpanzees and especially orangutans. PC2 (3.79% of variance) is strongly related to pollical distal phalanx length, and reveals a cline (from shorter to longer) with statistically significant differences between eastern and western gorillas (ANOVA; P=0.014), as well as between common and pygmy chimpanzees (P=0.047).

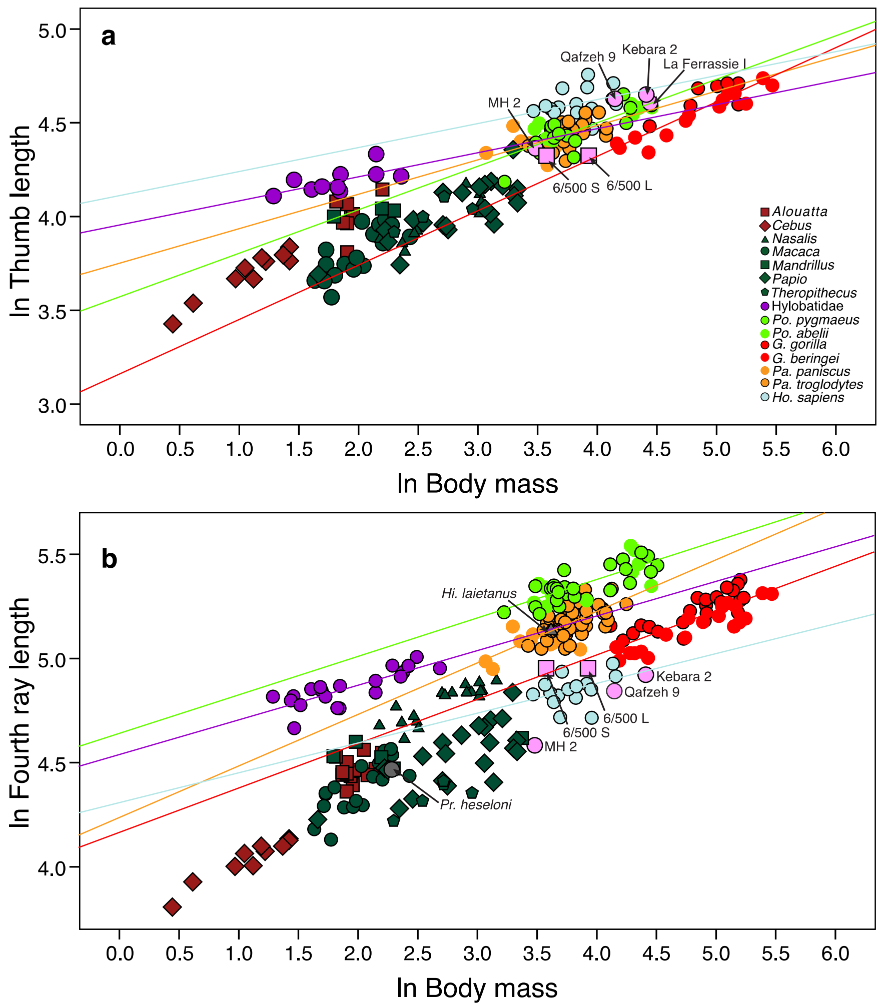

Supplementary Figure 4 | Allometric relationships of thumb (a) and fourth ray (b) lengths relative to body mass (BM) in humans and other anthropoid primates. In both cases, regression lines are fitted to hylobatids (purple), orangutans (light green), gorillas (red), chimpanzees (orange) and modern humans (light blue) and extended over the remaining comparative sample. Analyses of covariance (ANCOVA) show that there are not significant differences in the slopes of the thumb (F=1.954; P=0.107) and fourth ray (F=1.131; P=0.343) regressions. However, in both cases, modern humans show regression slopes that are not statistically different from zero (Supplementary Table 5), indicating that in humans thumb and digital lengths are not dependent on body size. Comparisons between pollical marginal means (evaluated at lnBM=3.796) reveal that modern humans and hylobatids (not statistically different) exhibit longer thumbs than great apes (P<0.001). Gorillas display even shorter thumbs (P=0.001) than chimpanzees and orangutans (the latter two showing no differences). Comparisons between fourth ray marginal means (evaluated at lnBM=3.828) reveal differences between each ape group and humans (P≤0.008), with the exception of chimpanzees and hylobatids (P=0.332). When accounting for allometric relationships, orangutans exhibit longer fourth ray than hylobatids/chimpanzees, which in turn, are longer than gorillas and humans respectively.

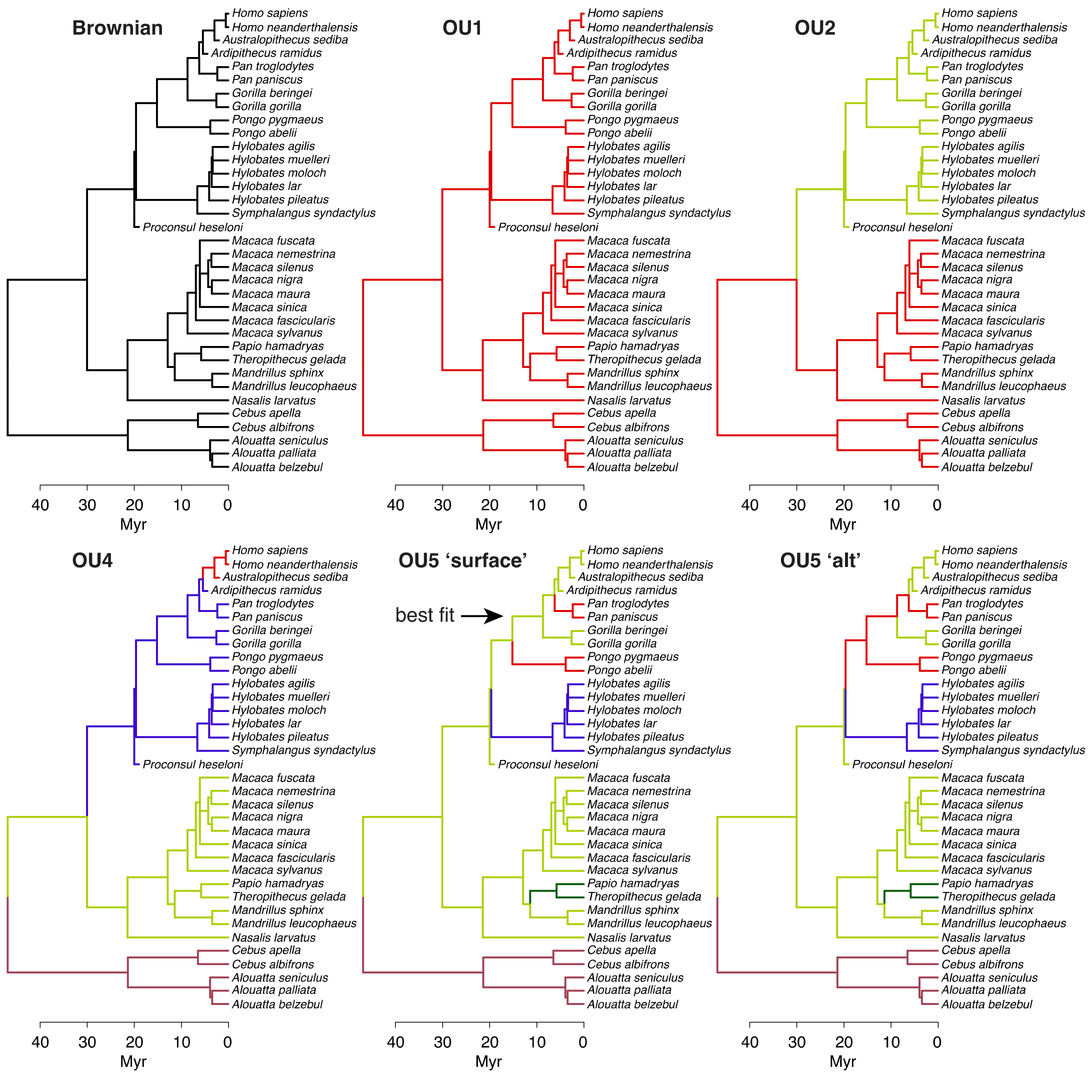

Supplementary Figure 5 | Alternative multivariate multi-regime Ornstein-Uhlenbeck (OU) hypotheses tested for the evolution of extrinsic hand proportions (EHP). Starting with Brownian motion as a baseline, we compared the relative fit (using AIC_c) of increasingly complex OU models with "ouch": one single regime (OU1), two regimes (OU2, hominoids vs. non-hominoids), four regimes (OU4; platyrrines, cercopithecids, non-human hominoids plus *Ardipithecus*, *Australopithecus-Homo*), the five regimes revealed by "surface" (OU5 'surface'; platyrrhines, *Papio-Theropithecus*, hylobatids, *Pan-Pongo*, rest of catarrhines). We further designed an alternative version of the previous model (OU5 'alt') in which *Pan* and *Pongo* were considered to reflect the plesiomorphic great ape condition. The OU5 'surface' model represented the best fit model, irrespective of the body mass estimate used for *Ardipithecus*, and the inclusion or not of *Ardipithecus* and *Proconsul* (see results in Supplementary Table 8).

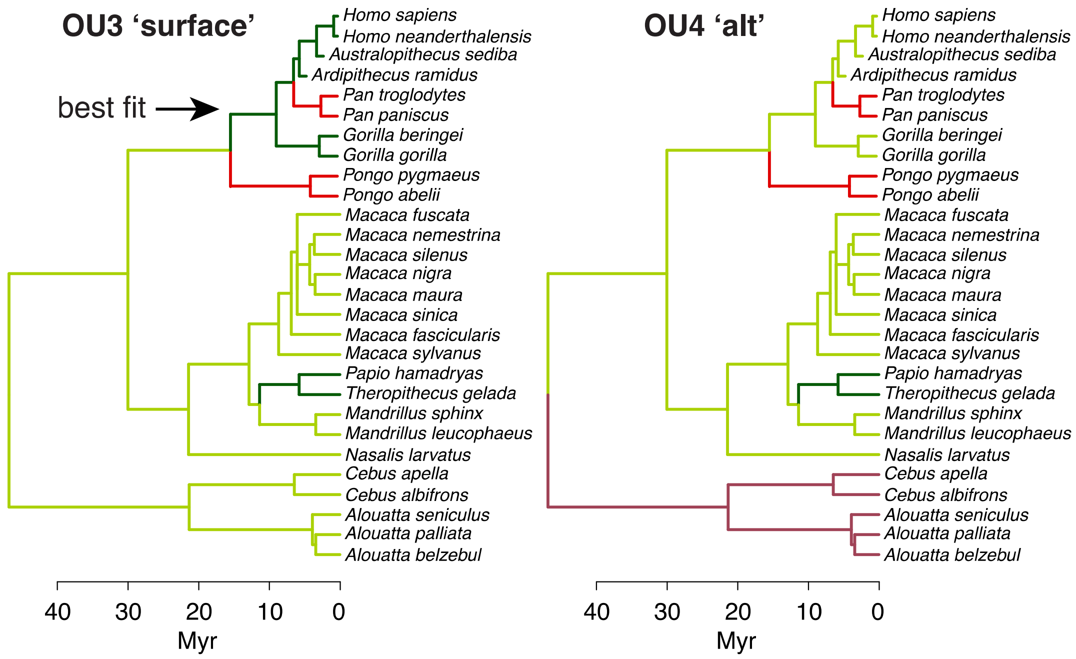

Supplementary Figure 6 | Sensitivity test of species sample size in alternative multivariate multi-regime Ornstein-Uhlenbeck (OU) models of extrinsic hand proportions evolution. We compared the fit of different OU models after dropping all hylobatid species and *Proconsul*. Alternative hypotheses included the OU1, OU2, and OU4 models described in Supplementary Figure 5, plus the three regime output revealed by "surface" (OU3 'surface'), and an alternative version of OU3 'surface' (OU4 'alt') based on the best fit model obtained for the full sample (Supplementary Fig. 5). In this case, the best fit model is represented by the OU3 'surface' output, in which upon an anthropoid regime baseline, hominins and gorillas share an optimum (convergent with baboons), whereas *Pan* and *Pongo* are again convergent (see results in Supplementary Table 8).

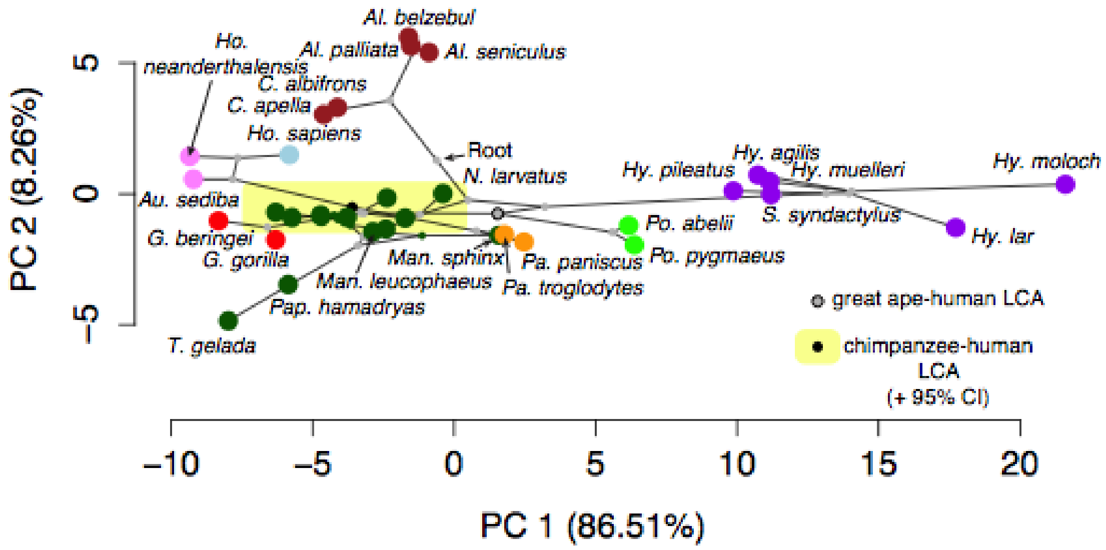

Supplementary Figure 7 | Reconstructed evolutionary history of human and ape hand proportions by excluding contentious fossils. Same approach as in Figure 4, but excluding *Proconsul heseloni* and *Ardipithecus ramidus*. Taxa are color-coded as in the phylogenetic tree (Fig. 3); internal nodes (i.e., ancestral-state reconstruction) are also indicated, highlighting the positions in shape-space of the great ape-human and chimpanzee-human LCA (plus 95% confident intervals for the latter estimate). The overall evolutionary pattern is comparable to that found in previous iterations including more fossils. Again, species of macaques were not labelled due to space restrictions.

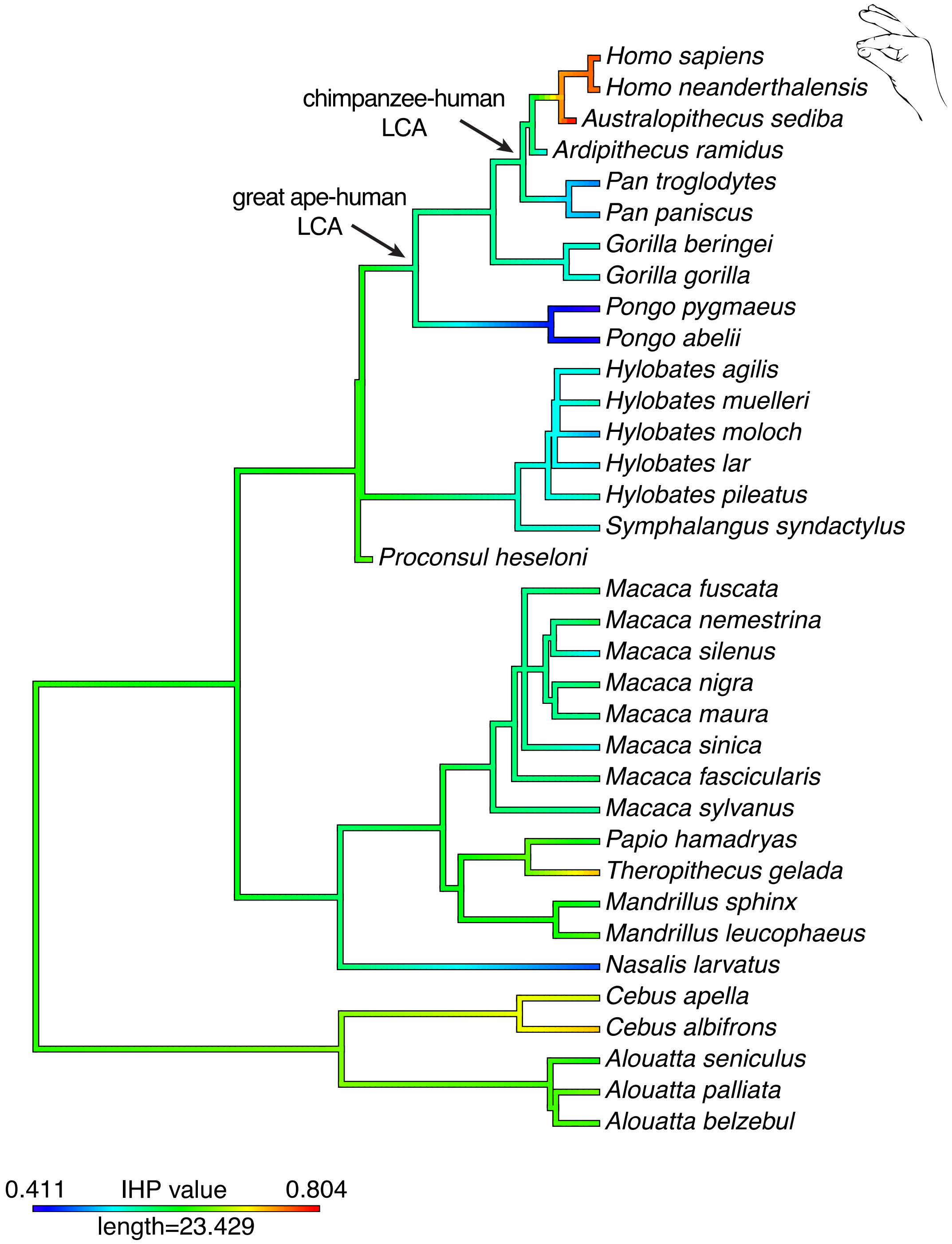

Supplementary Figure 8 | Evolution of intrinsic hand proportions (IHP) in humans and other anthropoid primates. The observed (Fig. 1) and reconstructed-state values are mapped along the branches and nodes of the anthropoid phylogeny. The ancestral state values for the great ape-human and chimpanzee-human last common ancestors (LCA) are highlighted with arrows. The IHP (relative long thumb) of humans, geladas and capuchin monkeys, as well as the IHP (different degrees of relative short thumb) of modern apes and *Nasalis* are reconstructed as having evolved (independently) from moderate proportions similar to *Proconsul*. Inset drawing represents a modern human performing a “pad-to-pad” precision grasping[1]. The length of the color legend at the bottom provides scale for the branches of the tree.

Supplementary Figure 8 | Continued.
This method to visualize trait evolution in a tree is explained in detail elsewhere[2]. Basically, ancestral characters are first estimated at the internal nodes again using ML[3,4] and Brownian motion[5-7]. Next, all edges along the tree are fractionated, and state estimates are computed at the midpoint of each fraction via interpolation using equation [3] of Felsenstein[7]. This creates the visual appearance of continuous color change along the edges of the tree.

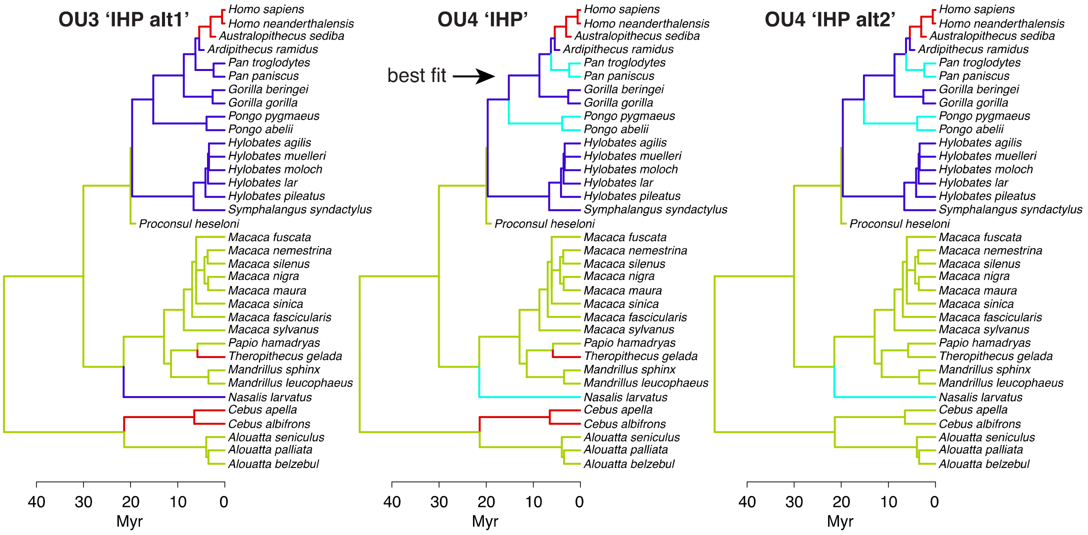

Supplementary Figure 9 | Alternative multi-regime Ornstein-Uhlenbeck (OU) hypotheses tested for the evolution of intrinsic hand proportions (IHP). In total, we compared the relative fit of eight different OU multi-regime models: Starting with Brownian motion, we followed with the increasingly complex models OU1, OU2, OU4, as well as the best fit model for extrinsic hand proportions described in Supplementary Figure 5. Furthermore we incorporated three extra models (this figure) based on the IHP results revealed by Figure 1 and Supplementary Figure 8 (as expected, "surface" did not perform well with univariate data[8]). The four adaptive regimes OU4 'IHP' represented the best fit model: *Australopithecus*-*Homo* share an adaptive regime with *Theropithecus* and *Cebus* (i.e., they are convergent for a relative long thumb); *Pan* and *Pongo* are convergent for a relative short thumb (as in the case of extrinsic hand proportions; Supplementary Fig. 5), as well as *Nasalis*; hylobatids, gorillas and *Ardipithecus* share the inferred plesiomorphic condition for crown apes, whereas the rest of the cercopithecid and platyrrhine monkeys share a more generalized regime. Alternative models in which *Pan*, *Pongo* and *Nasalis* share the same regime as other hominoids (OU3 'IHP alt1') or where *Theropithecus* and *Cebus* are not convergent with *Australopithecus*-*Homo* (OU4 'IHP alt2') exhibited an inferior fit (see results in Supplementary Table 8).

Supplementary Table 1 | Samples of extant primates used in each analysis. Non-hominoid sample continues in the following page.

taxon	N	species	IHP	EXP
Homo	**40**	*Ho. sapiens* [a]	40	15
Pan	**46**	*Pa. troglodytes* [b]	34	30
		Pa. paniscus [c]	12	10
Gorilla	**34**	*G. beringei* [d]	21	14
		G. gorilla [e]	13	7
Pongo	**27**	*Po. pygmaeus* [f]	19	15
		Po. abelii [g]	8	8
Hylobatidae	**14**	*Hy. agilis* [h]	2	1
		Hy. muelleri [h]	2	1
		Hy. moloch [h]	3	1
		Hy. lar [i]	4	4
		Hy. pileatus [j]	1	1
		S. syndactylus [k]	2	2

Supplementary Table 1 | Continued.

taxon	N	species	IHP	EXP
Macaca	**18**	*Ma. fuscata* [h]	2	2
		Ma. nemestrina [h]	4	4
		Ma. silenus [h]	2	2
		Ma. nigra [h]	2	2
		Ma. maura [h]	1	1
		Ma. sinica [h]	1	1
		Ma. fascicularis [h]	3	3
		Ma. sylvanus [h]	3	3
Papio	**50**	*Pap. hamadryas* [l]	50	22
Theropithecus	**5**	*T. gelada* [m]	5	4
Mandrillus	**3**	*Man. sphinx* [n]	2	2
		Man. leucophaeus [h]	1	1
Nasalis	**14**	*Nasalis larvatus* [i]	14	11
Cebus	**11**	*C. apella* [j]	3	3
		C. albifrons [o]	6	5
		C. sp. [o]	2	0
Alouatta	**8**	*Al. seniculus* [j]	5	5
		Al. palliata [h]	2	2
		Al. belzebul [h]	1	1
Ateles	**4**	*At. geoffroyi* [h]	0	2
		At. paniscus [h]	0	2
**Total**	**274**		**270**	**187**

Superscripts indicate the collection provenience for each taxon. (a) CMNH; (b) AMNH, Naturalis, RMCA, SBU; (c) RMCA, SBU; (d) AMNH, NRM, RMCA, USNM; (e) AMNH, PC; (f) AMNH, MCZ, Naturalis, USNM; (g) CMNH, Naturalis, USNM; (h) Naturalis; (i) MCZ, Naturalis; (j) AMNH; (k) AMNH, Naturalis; (l) AMNH, KNM, Naturalis, RMCA, SBU, USNM; (m) AMNH, NME, SBU; (n) Naturalis, RMCA; (o) AMNH, SBU.

Abbreviations: N (total sample size for genus), IHP (sample size for intrinsic hand proportions), EXP (sample size for extrinsic hand proportions), AMNH (American Museum of Natural History), CMNH (Cleveland Museum of Natural History), KNM (Kenya National Museums), MCZ (Museum of Comparative Zoology), Naturalis (Naturalis Biodiversity Center), NME (National Museum of Ethiopia), NRM (Swedish Museum of Natural History), PC (Powell-Cotton Museum), RMCA (Royal Museum of Central Africa), SBU (Stony Brook University), USNM (National Museum of Natural History).

Supplementary Table 2 | Intrinsic hand proportions. Bonferroni-corrected pairwise *post hoc* comparisons.

	Pa. paniscus	*Pa. troglodytes*	*G. gorilla*	*G. beringei*	*Po. abelii*	*Po. pygmaeus*	*Ho. sapiens*	Hylobatidae	*Papio*	*Theropithecus*	*Mandrillus*	*Macaca*	*Nasalis*	*Cebus*
Pa. troglodytes	1.000													
G. gorilla	**0.031**	**0.000**												
G. beringei	**0.000**	**0.000**	1.000											
Po. abelii	**0.000**	**0.000**	**0.000**	**0.000**										
Po. pygmaeus	**0.000**	**0.000**	**0.000**	**0.000**	1.000									
Ho. sapiens	**0.000**	**0.000**	**0.000**	**0.000**	**0.000**	**0.000**								
Hylobatidae	0.208	**0.000**	1.000	1.000	**0.000**	**0.000**	**0.000**							
Papio	**0.000**	**0.000**	**0.000**	**0.000**	**0.000**	**0.000**	**0.000**	**0.000**						
Theropithecus	**0.000**	**0.000**	**0.000**	**0.000**	**0.000**	**0.000**	**0.012**	**0.000**	**0.000**					
Mandrillus	**0.000**	**0.000**	**0.000**	**0.001**	**0.000**	**0.000**	**0.000**	**0.000**	1.000	**0.000**				
Macaca	**0.000**	**0.000**	**0.002**	0.269	**0.000**	**0.000**	**0.000**	**0.000**	0.597	**0.000**	0.520			
Nasalis	0.516	1.000	**0.000**	**0.000**	0.123	**0.000**	**0.000**	**0.000**	**0.000**	**0.000**	**0.000**	**0.000**		
Cebus	**0.000**	**0.000**	**0.000**	**0.000**	**0.000**	**0.000**	**0.000**	**0.000**	**0.000**	1.000	**0.000**	**0.000**	**0.000**	
Alouatta	**0.000**	**0.000**	**0.000**	**0.000**	**0.000**	**0.000**	**0.000**	**0.000**	**0.008**	**0.000**	1.000	**0.000**	**0.000**	**0.000**

Significant differences ($P<0.05$) are marked in bold.

Supplementary Table 3 | Results of the principal component analyses (PCA). Results are shown for the individuals-based extrinsic hand proportions in our full (Fig. 2A, Supplementary Fig. 2) and great apes only samples (Supplementary Fig. 3).

	**PCA full sample**			**PCA great apes**	
	**PC1**	**PC2**	**PC3**	**PC1**	**PC2**
**% var.**	79.77	10.49	6.69	92.38	3.79
**% var. cumulative**	79.77	90.26	96.95	92.38	96.17
**MC1L**	**0.66**	0.18	**-0.70**	**0.91**	0.13
**PP1L**	**0.51**	**0.80**	-0.23	**0.76**	0.41
**DP1L**	0.23	**0.70**	-0.15	0.38	**0.72**
**MC4L**	**0.95**	-0.30	-0.07	**0.99**	0.12
**PP4L**	**0.96**	0.18	0.19	**0.97**	-0.21
**IP4L**	**0.94**	0.22	0.22	**0.97**	-0.06

Data is provided only for the axes accounting for most of the variance, which are displayed in the plots. Loadings with absolute values ≥ 0.5 are marked in bold. Abbreviations: MC, metacarpal; PP, proximal phalanx; DP, distal phalanx; IP, intermediate phalanx; L, length. Each length was divided by the cube root of body mass.

Supplementary Table 4 | Extrinsic hand proportions. Bonferroni-corrected *post hoc* pairwise comparisons (Hotelling's p-values).

	Pa. paniscus	*Pa. troglodytes*	*G. gorilla*	*G. beringei*	*Po. abelii*	*Po. pygmaeus*	*Ho. sapiens*	Hylobatidae	*Papio*	*Theropithecus*	*Mandrillus*	*Macaca*	*Nasalis*	*Cebus*
Pa. troglodytes	1.000													
G. gorilla	**0.005**	**0.000**												
G. beringei	**0.000**	**0.000**	1.000											
Po. abelii	**0.005**	**0.000**	**0.000**	**0.000**										
Po. pygmaeus	**0.000**	**0.000**	**0.000**	**0.000**	1.000									
Ho. sapiens	**0.000**	**0.000**	**0.000**	**0.000**	**0.000**	**0.000**								
Hylobatidae	**0.000**	**0.000**	**0.000**	**0.000**	**0.000**	**0.000**	**0.000**							
Papio	**0.000**	**0.000**	**0.000**	**0.000**	**0.000**	**0.000**	**0.000**	**0.000**						
Theropithecus	**0.000**	**0.000**	**0.004**	**0.000**	**0.000**	**0.000**	**0.000**	**0.000**	**0.000**					
Mandrillus	**0.043**	**0.000**	0.508	**0.002**	**0.007**	**0.000**	**0.001**	**0.005**	**0.004**	1.000				
Macaca	**0.000**	**0.000**	**0.018**	**0.000**	**0.000**	**0.000**	**0.000**	**0.000**	**0.000**	**0.000**	0.234			
Nasalis	**0.001**	**0.000**	**0.001**	**0.000**	**0.001**	**0.000**	**0.000**	**0.000**	**0.000**	**0.000**	**0.002**	**0.000**		
Cebus	**0.000**	**0.000**	**0.000**	**0.000**	**0.000**	**0.000**	**0.000**	**0.000**	**0.000**	**0.000**	**0.002**	**0.000**	**0.000**	
Alouatta	**0.000**	**0.000**	**0.000**	**0.000**	**0.000**	**0.000**	**0.000**	**0.000**	**0.000**	**0.000**	**0.001**	**0.000**	**0.000**	**0.000**

Comparisons based in the three first principal components, which account for ~97% of the total variance. Significant differences ($P<0.05$) are marked in bold.

Supplementary Table 5 | Allometric regressions of thumb and fourth ray lengths (mm) relative to body mass (kg) in modern hominoids.

		N	R	SEE	p	slope	95% CI		intercept	95% CI	
**Thumb**	***Pan***	41	0.562	0.043	**0.000**	0.183	0.096	0.271	3.753	3.428	4.078
	Gorilla	21	0.897	0.033	**0.000**	0.29	0.221	0.359	3.162	2.827	3.497
	Pongo	23	0.809	0.037	**0.000**	0.232	0.156	0.308	3.573	3.278	3.869
	Homo	15	0.328	0.102	0.233	0.128	-0.093	0.349	4.113	3.271	4.955
	**Hylobatidae**	10	0.657	0.052	**0.039**	0.128	0.008	0.248	3.956	3.735	4.178
**Ray IV**	***Pan***	66	0.704	0.031	**0.000**	0.247	0.185	0.31	4.235	4.002	4.468
	Gorilla	44	0.801	0.025	**0.000**	0.213	0.163	0.262	4.164	3.926	4.403
	Pongo	36	0.776	0.026	**0.000**	0.185	0.132	0.237	4.64	4.436	4.844
	Homo	16	0.381	0.093	0.145	0.143	-0.056	0.342	4.308	3.551	5.065
	**Hylobatidae**	21	0.813	0.027	**0.000**	0.166	0.109	0.223	4.538	4.425	4.651

Significant slopes (i.e., statistically different from zero) are marked in bold. Humans are the only hominoids without predictable covariation between hand lengths and body size.

Supplementary Table 6 | Least-squares regressions of body mass (BM, kg) on femoral head diameter (FHD, mm). Regressions at the genus level in small modern humans and wild-shot primates.

	N	R	SEE	BM prediction
Pan (*troglodytes* and *paniscus*)	28	0.83	6.10	3.287 x FHD – 62.62
Gorilla (*gorilla* and *beringei*)	14	0.91	19.90	7.843 x FHD – 236.03
Pongo (*abelli* and *pygmaeus*)	19	0.94	7.90	5.265 x FHD – 123.63
Homo ("small humans")	*	0.88	2.17	1.747 x FHD – 24.602
**Hylobatidae** (*Hy. lar* and *S. syndactylus*)	18	0.93	1.10	1.176 x FHD – 12.96
Papio *hamadryas*	35	0.91	2.60	2.466 x FHD – 35.17
Macaca (*fascicularis* and *nemestrina*)	21	0.89	0.80	0.856 x FHD – 6.18
Nasalis *larvatus*	10	0.95	2.10	1.907 x FHD – 23.6
Cebus (*apella* and *albifrons*)	30	0.67	0.56	1.078 x FHD – 8.036
Alouatta (*seniculus* and *caraya*)	15	0.89	0.74	1.156 x FHD – 9.816

*The "small human" regression is based in ten sex-specific population means, including: Eastern and Western African pygmies, Khoe-San, Aeta and Andaman Islanders.

Supplementary Table 7 | Results of the principal component analyses (PCA) using the covariance matrix between species means. Results are provided for the full set of extrinsic hand proportions (Fig. 4a, b), and for the fourth ray only (Fig. 6a,b). Each analysis was iterated with a large (ArdiL; 50.8 kg) and small (ArdiS; 35.7 kg) body mass estimate for *Ar. ramidus*.

	**ArdiL**		**ArdiS**		**Hispano-ArdiL**		**Hispano-ArdiS**	
	**PC1**	**PC2**	**PC1**	**PC2**	**PC1**	**PC2**	**PC1**	**PC2**
**% var.**	86.34	8.18	86.21	8.30	92.83	6.88	92.75	6.96
**% var. cumulative**	86.34	94.52	86.21	94.51	92.83	99.71	92.75	99.71
**MC1L**	0.32	-0.15	0.31	-0.16				
**PP1L**	0.18	**0.53**	0.18	**0.53**				
**DP1L**	0.06	0.34	0.06	0.34				
**MC4L**	**0.66**	**-0.59**	**0.66**	**-0.59**	**0.70**	**0.71**	**0.70**	**0.71**
**PP4L**	**0.53**	0.39	**0.53**	0.39	**0.57**	**-0.57**	**0.57**	**-0.57**
**MP4L**	0.39	0.30	0.39	0.30	0.42	-0.42	0.42	-0.42

Data is provided only for the axes accounting for most of the variance, which are displayed in the plots. Loadings with absolute values ≥ 0.5 are marked in bold. Abbreviations: MC, metacarpal; PP, proximal phalanx; DP, distal phalanx; IP, intermediate phalanx; L, length. Each length was divided by the cube root of body mass.

Supplementary Table 8 | Results of alternative multivariate multi-regime Ornstein-Uhlenbeck (OU) hypothesis tests in 'ouch' for extrinsic (EHP) and intrinsic (IHP) hand proportions. For each model, we report a measure of relative model fit (ΔAIC_c) and support (Akaike weight[9]). The lowest ΔAIC_c score (0 in each case; indicated in bold) represents the best-fit model. Sensitivity analyses for EHP were iterated with a large (ArdiL; 50.8 kg) and small (ArdiS; 35.7 kg) body mass estimate for *Ar. ramidus*; as well as by excluding *Ar. ramidus* and *Proconsul heseloni* (NO-Ardi-Pro), and *Pr. heseloni* and all hylobatid species (NO-Pro-hylo) from the analysis.

	Model	AICc	ΔAIC_c	AIC_c weight
**EHP ArdiL**	Brownian	325.79	41.14	0.00
	OU1	329.86	45.21	0.00
	OU2	326.77	42.13	0.00
	OU4	316.70	32.06	0.00
	OU5 'surface'	**284.64**	**0.00**	**1.00**
	OU5 'alt'	299.51	14.87	0.00
**EHP ArdiS**	Brownian	328.24	43.59	0.00
	OU1	332.81	48.16	0.00
	OU2	329.36	44.72	0.00
	OU4	319.23	34.59	0.00
	OU5 'surface'	**284.64**	**0.00**	**1.00**
	OU5 'alt'	300.14	15.50	0.00
**EHP NO-Ardi-Pro**	Brownian	305.22	39.57	0.00
	OU1	314.61	48.96	0.00
	OU2	311.78	46.13	0.00
	OU4	294.13	28.49	0.00
	OU5 'surface'	**265.65**	**0.00**	**0.77**
	OU5 'alt'	268.05	2.41	0.23
**EHP NO-Pro-hylo**	Brownian	248.87	36.46	0.00
	OU1	252.39	39.98	0.00
	OU2	256.88	44.47	0.00
	OU4	245.74	33.33	0.00
	OU3 'surface'	**212.41**	**0.00**	**0.92**
	OU4 'alt'	217.34	4.93	0.08
**IHP**	Brownian	-68.98	56.85	0.00
	OU1	-71.86	53.97	0.00
	OU2	-72.52	53.31	0.00
	OU4	-96.50	29.32	0.00
	OU5 'EHP'	-82.24	43.59	0.00
	OU3 'IHP alt1'	-114.79	11.04	0.00
	OU4 'IHP'	**-125.83**	**0.00**	**1.00**
	OU4 'IHP alt2'	-108.96	16.87	0.00

See Supplementary Figures 5, 6, 9 for descriptions of each model.

Supplementary Note 1: What is "pad-to-pad" precision grasping?

Among the vast array of grips that the human and ape hand are capable of, Napier[10] defined the term "precision grip" to describe instances in which the object being manipulated (with precision) was held between the palmar aspects of the fingers and the opposing thumb. In contrast, "power grip" refers to situations in which the object is held in a "clamp fashion" between the flexed fingers and the palm, and the thumb only plays a subsidiary role by directing the force being applied (as when using a hammer). The term "human-like precision grasping" is commonly used in the literature although sometimes misunderstood: chimpanzees and orangutans can efficiently manipulate objects via different forms of precision grasping (e.g., thumb and index finger tip-to-tip and pad-to-side)[1,11,12]. However, the characteristic human "pad-to-pad" precision grip (i.e., flat contact between the proximal pulps of the thumb and one or more fingers)[13] is precluded in modern apes due to the disproportionate length of their digits II-V relative to the thumb[1,11,14,15] (Fig. 1), as well as by restricted passive hyperextension of the distal phalanges[12,15].

With the exception of hylobatids, a group that constitutes the exception to many rules in hand morphology[16,17], a clear trend is revealed within each anthropoid lineage: the more arboreal species exhibit functionally shorter thumbs relative to the fingers. As an example, within extant great apes the highly arboreal orangutans, followed by chimpanzees, display relatively shorter thumbs than the more terrestrial gorillas, which exhibit more generalized proportions (Fig. 1). This has been related to the capability of performing an effective "hook grasp" during below-branch suspension[1,11,16]. Among catarrhines, only *Theropithecus gelada* approaches the human condition in terms of IHP as computed in our analysis (Fig. 1), but since geladas exhibit an extremely shortened index finger[18], this "opposability index" would surpass the human condition if the index finger was the denominator instead of the fourth ray. The IHP in this species are explained as a specific feeding adaptation in primates that spend 70% of their daily activity collecting food (blades of grass, seeds and rhizomes) using precision grips[19]. The special adaptation of the hands of geladas is also evident in a special differentiation of the *flexor digitorium profundus*, as well as other thumb muscles[20], which is also reflected in their pollical distal phalanx morphology[17]. Capuchins (*Cebus*) monkeys are the only non-hominoid primates known to use tools habitually[21]. Although platyrrhines lack a "true opposable thumb"[11], capuchins (unlike other New World primates) commonly display both precision and power grips to manipulate objects such as use of stones as nut cracking tools, and stone flakes as cutting tools[22-24]. Thus, these behaviours are consistent with our results of intrinsic hand proportions (Fig.1), which we find to be convergent with humans (Supplementary Fig. 9).

Supplementary Note 2: Evolutionary scenarios supported by the results of this work

Our results show that contrarily to the idea assumed by some, extant great apes constitute a heterogeneous group in terms of hand and thumb proportions (Figs. 1-2, Supplementary Figs. 1-3). Furthermore, our evolutionary modelling unambiguously shows that the chimpanzee-human LCA exhibited a moderate hand length (relative to overall body size), more similar to humans than to chimpanzees (Figs. 3-4). Of special relevance is the fact that even using different phylogenetic hypotheses (Figs. 5-6, Supplementary Figs. 6-7) our results indicate that digital elongation has been achieved to different degrees and independently in the different extant and fossil ape lineages. Although the evidence presented here is restricted to the hand, broader implications can be reasonably drawn in terms of human and ape evolution:

1 - Mosaicism and Parallelism in Ape Evolution

Together with previous analyses of limb proportions[25] and skull morphology[26], these results falsify the view that extant apes, and particularly African apes constitute a homogeneous group with subtle deviations from a common allometric pattern[27]. Furthermore, the degree of heterogeneity in hand proportions revealed here is congruent with a mosaic evolution of the hominoid postcranial skeleton as inferred before from the fossil record[28-34]. Our results, indicating parallel evolution for digital elongation (with *Pan* and *Pongo* sharing convergent similarities, Fig. 3), match previous observations in other anatomical regions of modern apes[35-40]. In general, the current evidence reinforces the view that specialized arboreal adaptations exhibited by the living apes are not identical because they evolved independently as biomechanical solutions to largely similar but far from identical positional and locomotor behaviours[16,29,35], and parallelism was facilitated by their common genetic and developmental base[37,41]. One of the consequences of this hypothesis is that no extant ape will properly represent a living analogue for a given hypothetical ancestor[29,42].

2 - Extant Hominoids Are Survivors

As pointed out before, extant apes represent a very decimated expression of a highly diversified group during the Miocene[40,42-44]. What explains their decay? And why there are no fossil apes showing all the derived features of the living lineages? A possible explanation is that offered by Pilbeam and colleagues[45-47], who argue that we have not yet found any bona fide crown great ape in the fossil record. Another hypothesis that we favour is that a select few hominoid lineages (living representatives) survived *because* they were adapted to specialized lifestyles: e.g., enhanced antipronogrady and frugivory in hylobatids, orangutans and chimpanzees; large body size and folivory in gorillas; and finally, bipedalism and novel manual foraging strategies in hominins[14,48,49], and were able to compete with the radiation of the more generalized cercopithecids starting in the late Miocene[41,50]. If that were the

case, it is striking that the European late Miocene *Hispanopithecus/Rudapithecus* lineage (Fig. 5), with clearly specialized suspensory adaptations in the hand and other anatomical regions[28,30,34,51], became extinct at the end of the Miocene. It seems that in this case the specialized lifestyle that allowed the survival of most extant ape lineages became an evolutionary trap for *Hispanopithecus* during to the "Vallesian crisis" (ca. 9.5 Ma), which caused its extinction —as well as that of other forest-adapted fauna— as a consequence of paleoenvironmental changes associated with increased aridification and seasonality that caused the demise of the warm temperate forests (and year-round availability of fruit) in Western and Central Europe[52,53]. Furthermore, the results of this work indicate that suspensory behaviours in *Hispanopithecus laietanus* (as indicated by finger lengthening) evolved independently from other ape lineages (Fig. 6), reinforcing the view that the West European Miocene apes constituted an independent evolutionary radiation.

3 - Implications for Knuckle Walking

Humeral length relative to body mass is surprisingly similar in African apes and modern humans[54], but it is relatively longer in orangutans and lesser apes. These latter two suspensory hominoids also possess higher brachial indices (i.e., 100 x radius length/humerus length), whereas modern humans and gorillas have the lowest brachial indices among extant hominoids. *Proconsul*, *Ardipithecus* and australopiths (*Au. afarensis*, *Au. garhi*, and *Au. sediba*) all have intermediate brachial indices that overlap with chimpanzees[55], suggesting this to be the plesiomorphic proportionality for the upper limb of the African ape-human last common ancestor (LCA). However, our results on extrinsic hand proportions (EHP) favour the hypothesis that gorillas and early hominins are the most conservative in terms of overall hand shape (Figs. 3-4), in agreement with previous observations of Schultz[56]. This has implications for understanding the evolution of knuckle walking. Classically, the hands of great apes were seen as anatomical "hooks" designed for below-branch suspension, so they would be forced when on the ground to walk on the dorsal surfaces of their hooked hands[36,56]. However, this locomotor behaviour is currently seen as a compromise solution between the biomechanical requirements of advanced climbing and terrestrial digitigrady —requiring long versus short fingers, respectively[57,58]. Based on the terrestrial fist-walking of orangutans, Tuttle interpreted it as an intermediate stage between advanced arboreal suspension and terrestrial locomotion[15,58,59]. Whereas fist-walking allowed the hand to be used as a supporting structure, knuckle walking would further allow the manual phalanges to act as a propulsive lever during terrestrial quadrupedalism[58]. In our analyses (Fig. 4) the EHP of the African ape LCA are reconstructed as moderate in digital length (i.e., most similar to the chimpanzee-human LCA). Thus, irrespective of whether knuckle walking evolved only once at the base of the African ape lineage[45,57,60], or independently in gorillas and chimpanzees[55,58,61-63], our results imply that it was not related causally to the possession of especially long digits like those present in *Pan* or *Pongo*. Contrarily,

origins of knuckle walking should probably be interpreted only in the light of an adaptive complex that would reduce the compressive stresses, as well as the torques generated by the ground reaction force during hyperextension of the metacarpophalangeal joints during terrestrial quadrupedalism while still preserving a powerful grasping hand[64,65]. Among other bony features, it would be associated with: short phalanges relative to metacarpals, high dorsopalmar diameter of the metacarpal heads, as well as pronounced dorsal ridges and large epicondyles on the metacarpal heads. The question remains that if knuckle walking is such an efficient form of terrestrial quadrupedalism, why has it not evolved in other primates too? (as it has outside primates)[66]. The answer to this question is no doubt very complex; “regular” (monkey-like) digitigrady might be restricted by a certain threshold of absolute digital length and body mass within an ancestral terrestrial setting. Evolution of knuckle-walking was probably facilitated in African apes instead by their arboreal heritage[15]; having short tendons for the extrinsic flexor muscles is one of several limiting factors[12,15,67], and we hypothesize that the possession of an orthograde body plan, as well as long forelimbs relative to hindlimbs, combine to dictate the unusual way in which African apes can perform quadrupedalism.

4 - Implications for Early Hominin Locomotion

In relation to the longstanding debate on the climbing capabilities of early hominins[68-71], our results —showing similar digital length in gorillas and modern humans (Figs. 2, 4; Supplementary Fig. 1)— imply that in terms of digital length there is no reason to think that climbing behaviours observed in gorillas[72] were precluded in australopithecines. In fact, trained modern humans are excellent climbers[73], even exceeding gorillas in acrobatic capabilities[74]. Relevant to the origins of bipedalism, the preserved portions of the thorax and hand of the fossil great ape *Pierolapithecus* indicate that the acquisition of an orthograde body plan can be decoupled from specialized climbing and suspensory adaptations[29]. This evidence opens the possibility of human bipedalism having originated as a direct exaptation of arboreal orthogrady, without an intermediate stage of advanced suspension or specialized knuckle walking.

5 - Origins of the Human Hand

In terms of modern human hand proportions, most of the evolutionary change is concentrated in digital elongation/reduction (specifically metacarpal and proximal phalanx) whereas the thumb itself has remained more conservative, with just slight thumb elongation in humans (especially via proximal pollical phalanx; Supplementary Table 7). Therefore, within living apes (and anthropoids) modern humans do not exhibit the shortest hands nor the longest thumbs, but rather a useful combination that has been selected to allow enhanced thumb to fingers opposition (Fig. 1), as it is revealed by our convergence results with *Theropithecus* and *Cebus* (Supplementary

Fig. 9). Furthermore, these optimal intrinsic proportions evolved from a moderate ratio, as inferred for the chimpanzee-human LCA estimation, with less shape change than by assuming a chimpanzee-like LCA (Fig. 4, Supplementary Fig. 8). This confirms previous hypotheses based on observations of extant taxa, fossil apes and early hominins[17,31,63,75], and favors classic views of human evolution that preceded the molecular resolution of hominid phylogeny[15,56,67,76-78]. This and previous works indicate that enhanced thumb-to-digits opposition was present in australopiths *sensu lato*[14,49,79-84], but see Rolian and Gordon for a different opinion on *Au. afarensis*[85,86]. This would not be the case of the early Pliocene (4.4 Ma) *Ar. ramidus*, that exhibits a shorter thumb relative to fingers (i.e., IHP; in the gorilla and hylobatid range, but longer than chimpanzees; Fig. 1). However, evidence from the pollical distal phalanx morphology suggests that intrinsic hand proportions (IHP) similar to those of *Australopithecus*/humans (allowing for enhanced "pad-to-pad" opposition) could be already present in the late Miocene (ca. 6 Ma) *Orrorin tugenensis*[48,87], a hominin that was at least an incipient biped based on femoral morphology[40,88-92]. Since both extrinsic (Fig. 4) and intrinsic (Supplementary Fig. 8) hand proportions in *Ar. ramidus* seem largely plesiomorphic (for the African ape and human clade), this evidence suggests that although more recent in time than *O. tugenensis*, *Ar. ramidus* more closely reflects the hand proportions of the chimpanzee-human LCA[63]. If this were the case, this could represent a very early case of cladogenesis in the hominin lineage in which *Ar. ramidus* would be more plesiomorphic than *O. tugenensis*. A possible explanation for this would be niche partitioning in early hominins, with *Ar. ramidus* being more committed to arboreal life than *O. tugenensis*: Short thumbs relative to digits have almost always been related to arboreal locomotion[16,56,93], an environment for which *Ar. ramidus* was well suited in many other respects[63,94,95]. More fossils of *O. tugenensis* representing anatomies preserved in *Ar. ramidus* would be necessary to test this hypothesis.

A long thumb relative to fingers (i.e., high IHP; Fig. 1) facilitates enhanced pad-to-pad opposition and advanced manipulative skills in humans[1] and other non-hominoid primates[18,19,22-24]. But, did this high human IHP ratio evolve specifically for stone tool making? There is archaeological evidence indicating that stone tool use was part of the chimpanzee-human LCA behavioural repertoire[21,96], and thus not surprisingly also of *Au. afarensis*[97]. Furthermore, the thumb of *O. tugenensis* suggests human-like IHP at 6 Ma disassociated of stone tools[48]. We hypothesize that both human-like IHP and stone tool using behaviours evolved prior to the widespread appearance of systematic stone tool *making*, around 2.5 Ma[98,99], probably when the derived manual traits distinctive of modern humans and Neandertals first evolved[100,101]. More recently, the newly-described lithic artifacts from Lomekwi 3 (West Turkana, Kenya) push back the earliest evidence of intentional stone tool production at 3.3 Ma[102], which is consistent with human-like manual dexterity being an ancient adaptation amongst hominins. Harmand *et al.* argue that the decisive adaptation enabling "Lomekwian" stone knapping most likely related to a reorganization of the central nervous system in

yet unidentified hominins[102].

Thus, among the many features characterizing the human hand[1,10,11] such as a high IHP would not have necessarily evolved originally as a specific adaptation to stone tool making. Instead, they probably evolved as a new foraging/feeding adaptive complex, in the context of habitual bipedalism[14,48,74,103]. The relevance of bipedalism for the emergence of advanced manipulative skills in humans has been recognized ever since Darwin[104], but also in more recent works[14,74,105,106]. These authors share the same basic idea: regular bipedalism allowed some degree of relaxation of the locomotor selective pressures acting in the upper extremity, facilitating the manipulative selective pressures *already present in all primates*[1] to refine hand length proportions for advanced manipulative tasks. However, although foot-hand coevolution could have occurred via shared developmental pathways (i.e., pleiotropic effects)[107], our results indicate that these changes were relatively subtle (human manual hand proportions evolved from moderate —plesiomorphic— proportions, not from a chimp-like ancestor; Fig. 4 and Supplementary Fig. 8). Finally, we agree with idea that human hand length proportions are largely plesiomorphic for the hominin clade, and it was not until *later in time*, when these proportions were *co-opted*[108] for purposive and systematic stone tool making in hominins with more advanced cognitive capabilities[14,48,49,102,109].

Supplementary References

1 Napier, J. *Hands [Revised by Russell H. Tuttle].* 180 (Princeton University Press, 1993).

2 Revell, L. J. Two new graphical methods for mapping trait evolution on phylogenies. *Methods Ecol. Evol.* **4**, 754-759, (2013).

3 Felsenstein, J. Phylogenies and quantitative characters. *Annu. Rev. Ecol. Syst.* **19**, 445-471, (1988).

4 Schluter, D., Price, T., Mooers, A. Ø. & Ludwig, D. Likelihood of ancestor states in adaptive radiation. *Evolution* **51**, 1699-1711, (1997).

5 Cavalli-Sforza, L. L. & Edwards, A. W. Phylogenetic analysis. Models and estimation procedures. *Am. J. Hum. Genet.* **19**, 233, (1967).

6 Felsenstein, J. Maximum-likelihood estimation of evolutionary trees from continuous characters. *Am. J. Hum. Genet.* **25**, 471, (1973).

7 Felsenstein, J. Phylogenies and the comparative method. *Am. Nat.* **125**, 1-15, (1985).

8 Ingram, T. & Mahler, D. L. SURFACE: detecting convergent evolution from comparative data by fitting Ornstein - Uhlenbeck models with stepwise Akaike Information Criterion. *Methods Ecol. Evol.* **4**, 416-425, (2013).

9 Burnham, K. P. & Anderson, D. R. *Model Selection and Multimodel Inference: A Practical Information-theoretic Approach.* (Springer-Verlag, 2002).

10 Napier, J. R. The prehensile movements of the human hand. *J Bone Joint Surg Am.* **38 B**, 902-913, (1956).

11 Napier, J. R. Studies of the hands of living primates. *Proc. Zool. Soc. Lond.* **134**, 647-657, (1960).

12 Christel, M. in *Hands of primates* (eds Holger Preuschoft & David J. Chivers) 91-108 (Springer, 1993).

13 Shrewsbury, M. M., Marzke, M. W., Linscheid, R. L. & Reece, S. P. Comparative morphology of the pollical distal phalanx. *Am. J. Phys. Anthropol.* **121**, 30-47, (2003).

14 Alba, D. M., Moyà-Solà, S. & Köhler, M. Morphological affinities of the *Australopithecus afarensis* hand on the basis of manual proportions and relative thumb length. *J. Hum. Evol.* **44**, 225-254, (2003).

15 Tuttle, R. H. Knuckle-walking and the evolution of hominoid hands. *Am. J. Phys. Anthropol.* **26**, 171-206, (1967).

16 Straus, W. L., Jr. Rudimentary digits in primates. *Q. Rev. Biol.* **17**, 228-243, (1942).

17 Almécija, S., Shrewsbury, M., Rook, L. & Moyà-Solà, S. The morphology of *Oreopithecus bambolii* pollical distal phalanx. *Am. J. Phys. Anthropol.* **153**, 582-597, (2014).

18 Etter, H. F. Terrestrial adaptations in the hands of Cercopithecinae. *Folia Primatol.* **20**, 331-350, (1973).

19 Jolly, C. J. in *Diverse Approaches in Human Evolution* Vol. 4 323-332 (1970).

20 Maier, W. Vergleichende und funktionell-anatomische Untersuchungen an der Vorderextremitaet von *Theropithecus gelada* (Rueppell 1835). *Abh Senckenb naturforsch Ges* **527**, 1-284, (1971).

21 Panger, M. A., Brooks, A. S., Richmond, B. G. & Wood, B. Older than the Oldowan? Rethinking the emergence of hominin tool use. *Evol. Anthropol.* **11**, 235-245, (2002).
22 Costello, M. B. & Fragaszy, D. M. Prehension in *Cebus* and *Saimiri*: I. Grip type and hand preference. *Am. J. Primatol.* **15**, 235-245, (1988).
23 Westergaard, G. C. & Suomi, S. J. Capuchin monkey (*Cebus apella*) grips for the use of stone tools. *Am. J. Phys. Anthropol.* **103**, 131-135, (1997).
24 Marzke, M. W. Tool making, hand morphology and fossil hominins. *Philos. T. Roy. Soc. B* **368**, 20120414, (2013).
25 Jungers, W. L. & Hartman, S. E. in *Orang-utan Biology* (ed Jeffrey H. Schwartz) 347-359 (Oxford University Press, 1988).
26 Mitteroecker, P., Gunz, P., Bernhard, M., Schaefer, K. & Bookstein, F. L. Comparison of cranial ontogenetic trajectories among great apes and humans. *J. Hum. Evol.* **46**, 679-698, (2004).
27 Shea, B. T. Allometry and heterochrony in the African apes. *Am. J. Phys. Anthropol.* **62**, 275-289, (1983).
28 Moyà-Solà, S. & Köhler, M. A *Dryopithecus* skeleton and the origins of great-ape locomotion. *Nature* **379**, 156-159, (1996).
29 Moyà-Solà, S., Köhler, M., Alba, D. M., Casanovas-Vilar, I. & Galindo, J. *Pierolapithecus catalaunicus*, a new Middle Miocene great ape from Spain. *Science* **306**, 1339-1344, (2004).
30 Almécija, S., Alba, D. M., Moyà-Solà, S. & Köhler, M. Orang-like manual adaptations in the fossil hominoid *Hispanopithecus laietanus*: first steps towards great ape suspensory behaviours. *P. Roy. Soc. B* **274**, 2375-2384, (2007).
31 Almécija, S., Alba, D. M. & Moyà-Solà, S. *Pierolapithecus* and the functional morphology of Miocene ape hand phalanges: paleobiological and evolutionary implications. *J. Hum. Evol.* **57**, 284-297, (2009).
32 Hammond, A. S., Alba, D. M., Almécija, S. & Moyà-Solà, S. Middle Miocene *Pierolapithecus* provides a first glimpse into early hominid pelvic morphology. *J. Hum. Evol.* **64**, 658-666, (2013).
33 Tallman, M., Almécija, S., Reber, S. L., Alba, D. M. & Moyà-Solà, S. The distal tibia of *Hispanopithecus laietanus*: More evidence for mosaic evolution in Miocene apes. *J. Hum. Evol.* **64**, 319-327, (2013).
34 Susanna, I., Alba, D. M., Almécija, S. & Moyà-Solà, S. The vertebral remains of the late Miocene great ape *Hispanopithecus laietanus* from Can Llobateres 2 (Vallès-Penedès Basin, NE Iberian Peninsula). *J. Hum. Evol.* **73**, 15-34, (2014).
35 Larson, S. G. Parallel evolution in the hominoid trunk and forelimb. *Evol. Anthropol.* **6**, 87-99, (1998).
36 Erikson, G. E. Brachiation in New World monkeys and in anthropoid apes. *Symp. Zool. Soc. Lond.* **10**, 135-163, (1963).
37 Tuttle, R. in *Phylogeny of the Primates* (eds W Patrick Luckett & Frederick S Szalay) 447-480 (Springer, 1975).
38 Ward, C. in *Handbook of Paleoanthropology* (eds W. Henke & I. Tattersall) 1011-1030 (Springer Verlag, 2007).
39 Kivell, T., Barros, A. & Smaers, J. Different evolutionary pathways underlie the morphology of wrist bones in hominoids. *BMC Evol Biol* **13**, 229, (2013).

40 Almécija, S. *et al.* The femur of *Orrorin tugenensis* exhibits morphometric affinities with both Miocene apes and later hominins. *Nat. Commun.* **4**, 2888, (2013).
41 Reno, P. L. Genetic and developmental basis for parallel evolution and its significance for hominoid evolution. *Evol. Anthropol.* **23**, 188-200, (2014).
42 Harrison, T. The implications of *Oreopithecus bambolii* for the origins of bipedalism. *Origine(s) de la bipédie chez les hominidés*, 235-244, (1991).
43 Alba, D. M. Fossil apes from the Vallès-Penedès Basin. *Evol. Anthropol.* **21**, 254-269, (2012).
44 Begun, D. R., Nargolwalla, M. C. & Kordos, L. European Miocene hominids and the origin of the African ape and human clade. *Evol. Anthropol.* **21**, 10-23, (2012).
45 Pilbeam, D. & Young, N. Hominoid evolution: synthesizing disparate data. *C. R. Palevol* **3**, 305-321, (2004).
46 Pilbeam, D. R., Rose, M. D., Barry, J. C. & Shah, S. M. I. New *Sivapithecus* humeri from Pakistan and the relationship of *Sivapithecus* and *Pongo*. *Nature* **348**, 237-239, (1990).
47 Morgan, M. E. *et al.* A partial hominoid innominate from the Miocene of Pakistan: Description and preliminary analyses. *P. Natl Acad. Sci. USA* **Early Edition**, (2014).
48 Almécija, S., Moyà-Solà, S. & Alba, D. M. Early origin for human-like precision grasping: A comparative study of pollical distal phalanges in fossil hominins. *PLoS ONE* **5**, e11727, (2010).
49 Almécija, S. & Alba, D. M. On manual proportions and pad-to-pad precision grasping in *Australopithecus afarensis*. *J. Hum. Evol.* **73**, 88-92, (2014).
50 Jablonski, N. G., Whitfort, M. J., Roberts-Smith, N. & Qinqi, X. The influence of life history and diet on the distribution of catarrhine primates during the Pleistocene in eastern Asia. *J. Hum. Evol.* **39**, 131-157, (2000).
51 Pina, M., Alba, D. M., Almécija, S., Fortuny, J. & Moyà-Solà, S. Brief communication: Paleobiological inferences on the locomotor repertoire of extinct hominoids based on femoral neck cortical thickness: The fossil great ape *Hispanopithecus laietanus* as a test-case study. *Am. J. Phys. Anthropol.* **149**, 142-148, (2012).
52 DeMiguel, D., Alba, D. M. & Moyà-Solà, S. Dietary specialization during the evolution of Western Eurasian hominoids and the extinction of European great apes. *PLoS ONE* **9**, e97442, (2014).
53 Casanovas-Vilar, I., Alba, D. M., Garcés, M., Robles, J. M. & Moyà-Solà, S. Updated chronology for the Miocene hominoid radiation in Western Eurasia. *P. Natl Acad. Sci. USA* **108**, 5554-5559 (2011).
54 Jungers, W. L. Ape and hominid limb length. *Nature* **369**, 194-194, (1994).
55 Lovejoy, C. O., Suwa, G., Simpson, S. W., Matternes, J. H. & White, T. D. The great divides: *Ardipithecus ramidus* reveals the postcrania of our last common ancestors with African apes. *Science* **326**, 73-106, (2009).
56 Schultz, A. H. The skeleton of the trunk and limbs of higher primates. *Hum. Biol.* **2**, 303-438, (1930).
57 Richmond, B. G., Begun, D. R. & Strait, D. S. Origin of human bipedalism: the knuckle-walking hypothesis revisited. *Yearb. Phys. Anthropol.* **44**, 70-105, (2001).
58 Tuttle, R. H. Knuckle-walking and the problem of human origins. *Science* **166**, 953-961, (1969).

59 Tuttle, R. Knuckle walking hand postures in an orangutan (*Pongo pygmaeus*). *Nature* **236**, 33-34, (1972).
60 Williams, S. A. Morphological integration and the evolution of knuckle-walking. *J. Hum. Evol.* **58**, 432-440, (2010).
61 Dainton, M. & Macho, G. A. Did knuckle walking evolve twice? *J. Hum. Evol.* **36**, 171-194, (1999).
62 Kivell, T. L. & Schmitt, D. Independent evolution of knuckle-walking in African apes shows that humans did not evolve from a knuckle-walking ancestor. *P. Natl Acad. Sci. USA* **Early Edition**, (2009).
63 Lovejoy, C. O., Simpson, S. W., White, T. D., Asfaw, B. & Suwa, G. Careful climbing in the Miocene: The forelimbs of *Ardipithecus ramidus* and humans are primitive. *Science* **326**, 70-708, (2009).
64 Preuschoft, H. Functional anatomy of the upper extremity. *The Chimpanzee* **6**, 34-120, (1973).
65 Susman, R. L. Comparative and functional morphology of hominoid fingers. *Am. J. Phys. Anthropol.* **50**, 215-236, (1979).
66 Orr, C. M. Knuckle-walking anteater: A convergence test of adaption for purported knuckle-walking features of African Hominidae. *Am. J. Phys. Anthropol.*, (2005).
67 Straus, W. L., Jr. The posture of the great ape hand in locomotion, and its phylogenetic implications. *Am. J. Phys. Anthropol.* **27**, 199-207, (1940).
68 Johanson, D. C. *et al.* Morphology of the Pliocene partial hominid skeleton (A.L. 288-1) from the Hadar Formation, Ethiopia. *Am. J. Phys. Anthropol.* **57**, 403-451, (1982).
69 Stern, J. T., Jr. & Susman, R. L. The locomotor anatomy of *Australopithecus afarensis*. *Am. J. Phys. Anthropol.* **60**, 279-317, (1983).
70 Stern, J. T. J. Climbing to the top: a personal memoir of *Australopithecus afarensis*. *Evol. Anthropol.* **9**, 113-133, (2000).
71 Ward, C. V., Kimbel, W. H. & Johanson, D. C. Complete fourth metatarsal and arches in the foot of *Australopithecus afarensis*. *Science* **331**, 750-753, (2011).
72 Remis, M. Effects of body size and social context on the arboreal activities of lowland gorillas in the Central African Republic. *Am. J. Phys. Anthropol.* **97**, 413-433, (1995).
73 Venkataraman, V. V., Kraft, T. S. & Dominy, N. J. Tree climbing and human evolution. *P. Natl Acad. Sci. USA*, (2012).
74 Hewes, G. W. Food transport and the origin of hominid bipedalism. *Am. Anthropol.* **63**, 687-710, (1961).
75 Almécija, S., Alba, D. M. & Moyà-Solà, S. The thumb of Miocene apes: New insights from Castell de Barberà (Catalonia, Spain). *Am. J. Phys. Anthropol.* **148**, 436-450, (2012).
76 Straus, W. L., Jr. The riddle of man's ancestry. *Q. Rev. Biol.* **24**, 200-223, (1949).
77 Schultz, A. H. Characters common to higher primates and characters specific for man. *Q. Rev. Biol.* **11**, 259-283, (1936).
78 Le Gros Clark, W. E. *The Fossil Evidence for Human Evolution.* 200 (University of Chicago Press, 1964).
79 Kivell, T. L., Kibii, J. M., Churchill, S. E., Schmid, P. & Berger, L. R. *Australopithecus sediba* hand demonstrates mosaic evolution of locomotor and manipulative abilities. *Science* **333**, 1411-1417, (2011).

80 Green, D. J. & Gordon, A. Metacarpal proportions in *Australopithecus africanus*. *J. Hum. Evol.* **54**, 705-719, (2008).
81 Marzke, M. W. Joint functions and grips of the *Australopithecus afarensis* hand, with special reference to the region of capitate. *J. Hum. Evol.* **12**, 197-211, (1983).
82 Susman, R. L. Hand of *Paranthopus robustus* from member 1, Swartkrans: Fossil evidence for tool behavior. *Science* **240**, 781-784, (1988).
83 Susman, R. L. Fossil evidence for early hominid tool use. *Science* **265**, 1570-1573, (1994).
84 Skinner, M. M. *et al.* Human-like hand use in *Australopithecus africanus*. *Science* **347**, 395-399, (2015).
85 Rolian, C. & Gordon, A. D. Reassessing manual proportions in *Australopithecus afarensis*. *Am. J. Phys. Anthropol.* **152** 393-406, (2013).
86 Rolian, C. & Gordon, A. D. Response to Almécija and Alba (2014) – On manual proportions in *Australopithecus afarensis*. *J. Hum. Evol.* **73**, 93-97, (2014).
87 Gommery, D. & Senut, B. La phalange distale du pouce d'*Orrorin tugenensis* (Miocène supérieur du Kenya). *Geobios* **39**, 372-284, (2006).
88 Pickford, M., Senut, B., Gommery, D. & Treil, J. Bipedalism in *Orrorin tugenensis* revealed by its femora. *C. R. Palevol* **1**, 191-203, (2002).
89 Galik, K. *et al.* External and internal morphology of the BAR 1002'00 *Orrorin tugenensis* femur. *Science* **305**, 1450-1453, (2004).
90 Richmond, B. G. & Jungers, W. L. *Orrorin tugenensis* femoral morphology and the evolution of hominin bipedalism. *Science* **319**, 1662-1665, (2008).
91 Richmond, B. G. & Jungers, W. L. in *African Genesis: Perspectives on Hominin Evolution* (eds Sally C. Reynolds & Andrew Gallagher) 248-267 (Cambridge University Press, 2012).
92 Senut, B., Pickford, M., Gommery, D. & Kunimatsu, Y. Un nouveau genge d'hominoïde du Miocène inférieur d'Afrique orientale: *Ugandapithecus major* (Le Gros Clark & Leakey, 1950). *C. R. Acad. Sci. Paris* **331**, 227-233, (2000).
93 Ashley-Montagu, F. M. On the primate thumb. *Am. J. Phys. Anthropol.* **25**, 291-314, (1931).
94 White, T. D. *et al.* *Ardipithecus ramidus* and the paleobiology of early hominids. *Science* **326**, 64-86, (2009).
95 Lovejoy, C. O., Latimer, B., Suwa, G., Asfaw, B. & White, T. D. Combining prehension and propulsion: The foot of *Ardipithecus ramidus*. *Science* **326**, 72-728, (2009).
96 Mercader, J., Panger, M. & Boesch, C. Excavation of a chimpanzee stone tool site in the African rainforest. *Science* **296**, 1452-1455, (2002).
97 McPherron, S. P. *et al.* Evidence for stone-tool-assisted consumption of animal tissues before 3.39 million years ago at Dikika, Ethiopia. *Nature* **466**, 857-860, (2010).
98 Semaw, S. *et al.* 2.5-million-year-old stone tools from Gona, Ethiopia. *Nature* **385**, 333-336, (1997).
99 Semaw, S. *et al.* 2.6-Million-year-old stone tools and associated bones from OGS-6 and OGS-7, Gona, Afar, Ethiopia. *J. Hum. Evol.* **45**, 169-177, (2003).
100 Tocheri, M. W., Orr, C. M., Jacofsky, M. C. & Marzke, M. W. The evolutionary history of the hominin hand since the last common ancestor of *Pan* and *Homo*. *J. Anat.* **212**, 544-562, (2008).

101 Ward, C. V., Tocheri, M. W., Plavcan, J. M., Brown, F. H. & Manthi, F. K. Early Pleistocene third metacarpal from Kenya and the evolution of modern human-like hand morphology. *P. Natl Acad. Sci. USA* **111**, 121-124, (2014).
102 Harmand, S. *et al.* 3.3-million-year-old stone tools from Lomekwi 3, West Turkana, Kenya. *Nature* **521**, 310-315, (2015).
103 Hunt, K. D. The postural feeding hypothesis: An ecological model for the evolution of bipedalism. *S Afr J Sci* **92**, 77, (1996).
104 Darwin, C. *The Descent of Man, and Selection in Relation to Sex*. (John Murray, 1871).
105 Hartwig, W. C. & Doneski, K. Evolution of the hominid hand and tool making behavior. *Am. J. Phys. Anthropol.* **106**, 401-402, (1998).
106 Jouffroy, F. K. in *Origine(s) de la Bipédie Chez les Hominidés* (eds B. Senut & Y. Coppens) 21-35 (Editions du CNRS, 1991).
107 Rolian, C., Lieberman, D. E. & Hallgrímsson, B. The coevolution of human hands and feet. *Evolution* **64**, 1558-1568, (2010).
108 Gould, S. J. & Vrba, E. S. Exaptation-a missing term in the science of form. *Paleobiology* **8**, 4-15, (1982).
109 Napier, J. Fossil hand bones from Olduvai Gorge. *Nature* **196**, 409-411, (1962).
